# Secretory Phospholipases A_2_ in Plants

**DOI:** 10.3389/fpls.2019.00861

**Published:** 2019-07-10

**Authors:** María Elisa Mariani, Gerardo Daniel Fidelio

**Affiliations:** ^1^Departamento de Química Biológica, Facultad de Ciencias Exactas, Físicas y Naturales, Universidad Nacional de Córdoba, Córdoba, Argentina; ^2^Instituto de Investigaciones Biológicas y Tecnológicas, Consejo Nacional de Investigaciones Científicas y Técnicas, Universidad Nacional de Córdoba, Córdoba, Argentina; ^3^Departamento de Fundamentación Biológica, Facultad de Ciencias Agropecuarias, Universidad Nacional de Córdoba, Córdoba, Argentina; ^4^Departamento de Química Biológica, Facultad de Ciencias Químicas, Universidad Nacional de Córdoba, Córdoba, Argentina; ^5^Centro de Investigaciones en Química Biológica de Córdoba, Consejo Nacional de Investigaciones Científicas y Técnicas, Universidad Nacional de Córdoba, Córdoba, Argentina

**Keywords:** secretory phospholipase A_2_, interfacial catalysis, auxin, *Glycine max*, phospholipase–membrane interaction

## Abstract

Secreted phospholipases (sPLA_2_s) in plants are a growing group of enzymes that catalyze the hydrolysis of *sn-2* glycerophospholipids to lysophospholipids and free fatty acids. Until today, around only 20 sPLA_2_s were reported from plants. This review discusses the newly acquired information on plant sPLA_2_s including molecular, biochemical, catalytic, and functional aspects. The comparative analysis also includes phylogenetic, evolutionary, and tridimensional structure. The observations with emphasis in *Glycine max* sPLA_2_ are compared with the available data reported for all plants sPLA_2_s and with those described for animals (mainly from pancreatic juice and venoms sources).

## Introduction

For more than a century experiments were performed with sPLA_2_s enzymes, being used as lipid model enzymology and as paradigms for the formalism of interfacial catalysis (Dennis et al., 2011). The phospholipase A_2_ (PLA_2_, EC 3.1.1.4) superfamily is a broad and growing group of enzymes that stereo specifically catalyzes the cleavage at the *sn-2* acyl ester bond from diacyl-phospholipid liberating lysophospholipid and free fatty acid. In plants, secreted PLA_2_ (sPLA_2_) represents one type of phospholipase A_2_ whose lipid products mediate a variety of cellular processes, including growth, development, defense, and stress responses (Stahl et al., 1998, 1999; Kim et al., 1999; Lee et al., 2003; Ryu, 2004; Mansfeld, 2009; Chen et al., 2011). Although numerous sPLA_2_ genes have been identified in plants, little is known about these enzymes in opposition to their insect, animal or human counterparts (Burke and Dennis, 2009). sPLA_2_ is best known from mammals where several sPLA_2_s have been identified in the last 25 years (Murakami et al., 2011). Moreover, many sPLA_2_s were found in sources as venoms from snakes, scorpions, bee, etc.; from microorganisms as bacteria and yeasts, as components of pancreatic juices, where it occurs abundantly and has a digestive role; arthritic synovial fluid; and in many different mammalian tissues (Valentin et al., 1999; Schaloske and Dennis, 2006; Burke and Dennis, 2009; Murakami et al., 2010, 2011). Additionally, for the first time, we have recently described the interfacial properties of purified recombinant sPLA_2_s from *Streptomyces violaceoruber* (Yunes Quartino et al., 2015) and from *Glycine max* (Mariani et al., 2012, 2015b), i.e., the optimal surface lipid packing conditions (interfacial quality) in which a sPLA_2_ can hydrolyze phospholipid in an organized membrane. This point, no less important for interfacial enzymes, was also addressed comparatively in the present review.

*Glycine max* (Soybean), in addition to being one of the most widely used oil crop grain in the world, possesses valuable contributions to health due to its high nutritional level. Lipids, proteins and other valuable bioactive components such as: phospholipids (known as lecithin), hormones, and antioxidants are present in soybean (Messina, 1999; Choi and Rhee, 2006). The industrial use of sPLA_2_s from animal pancreas extracts and microbes, especially in food production, has a long tradition (Guo et al., 2005; De Maria et al., 2007). One of the targets in the future may be the utilization of sPLA_2_ from plants for enzymatic processing to stereospecifically obtain lysoderivatives. This alternative has been recently recognized to satisfy food regulation requirements such as Kosher and Halal (Havinga, 2010). However, no sPLA_2_s from plants have now been yet available for industrial application (Mansfeld, 2009).

Secreted PLA_2_s are low MW calcium dependent enzymes (12–18 kDa) (Schaloske and Dennis, 2006). From a perusal revision of sequence data, almost all sPLA_2_s from plants and animals contain a signal sequence. So, in the general secretion way after removal of the N-terminal signal peptide in the endoplasmic reticulum (ER), they are secreted into the extracellular space in a either mature or pre-protein form (Fujikawa et al., 2005; Lee et al., 2005; Mansfeld et al., 2006). Although sPLA_2_s are recognized to be secreted proteins, a few of them were reported to act intracellularly prior or during secretion (Mounier et al., 2004; Shridas and Webb, 2014). Until now, the pre-protein form would be exclusive for animals (see Table 1).

**TABLE 1:** General characteristics presented by calcium dependent sPLA_2_s from animals to plants.

**Properties/characteristics**	**Animals**	**Plants**
Intracellular second messenger	PL → arachidonic acid → prostaglandins and leukotrienes	PL → linoleic acid → jasmonic acid
Main metabolic pathway	Eicosanoid pathway	Octadecanoid pathway
Secreted as zymogen	Some	NR
Catalytic triad	ASP/HIS/ASP	ASP/HIS/X (X = ASN or SER or HIS)
MW (kDa)	12–18	∼14
Cysteines		
Disulphide bridges	8–14	
4–7	12	
6		
Calcium requirement^a^	mM^b^	μM-mM

*PL, phospholipid; NR, no reported. ^*a*^Minimum of required Ca^2+^ concentration for full activity. ^*b*^Regarding to the general mM requirement for reported sPLA_2_ from animal source, it has been described one exception for a sPLA_2_ isolated from venom of the marine snail *Conus magus* (McIntosh et al., 1995). This exception for sPLA_2_ was also remarked by Six and Dennis (2000).*

Important common features shared for all sPLA_2_s are the presence of: (i) one HIS residue at the catalytic domain for nucleophilic attack at the *sn-2* acyl ester bond of the glycerol backbone, (ii) requisite of calcium for full activity (μM-mM), and (iii) exceptionally heat-stable enzymes. sPLA_2_s also contain a domain, named PA_2_c, with the highly conserved Ca^2+^ binding loop (YGKYCGxxxxGC) and the active site motif (DACCxxHDxC), where the HIS/ASP pair was found to be highly well conserved in both animal and plants sPLA_2_s. At least, two characteristics are of great interest in the structure of all sPLA_2_s: the catalytic site and the interfacial recognition surface (IRS). All sPLA_2_s have the same architecture (about 55% of identity) at the catalytic site level (HIS-ASP) (Lee et al., 2005) but differ in the amino acid residues that conform the IRS region (Berg et al., 2001) sharing only 15% of identity in the amino acid sequence.

In the presence of reducing compounds such as β-mercaptoethanol or dithiothreitol (DTT) the activity is affected or abolished by disrupting the protein structure (reduction of disulfide bridges) (Stahl et al., 1998). They also show high resistance to organic solvents, acidic conditions and high temperatures (they are even more resistant in the presence of Ca^2+^). A common procedure to confirm the catalytic mechanism is by checking if the activity is chemically canceled by the alkylation of HIS localized in the catalytic triad HIS/ASP/X (where X may be either HIS, SER, or ASP) induced by p-bromophenacylbromide (BPB) (Minchiotti et al., 2008). A resume of the general characteristics comparing animals from plants sPLA_2_ is shown in Table 1.

Fatty acids produced by the hydrolysis carried out by sPLA_2_s, such as oleic (1:18) or arachidonic (4:20) acid, are sources of energy reserve. Furthermore, arachidonic acid can function as intracellular second messenger or as precursor of eicosanoids inflammation mediators, if is the extracellular product of the reaction catalyzed by secreted phospholipase as occurs for human synovial fluid (Baynes and Marek, 2004). The other product of the action of sPLA_2_, the lysophospholipid is important in cell signaling and remodeling or membrane perturbations (Khan et al., 1995). In contrast, in plants the jasmonic acid and its related compounds are important hormones involved in plant defense reaction against microbial pathogens, herbivores and UV light damaging as well as senescence mechano-transduction (Schaller, 2001).

In the past years, significant advances have been made toward understanding the role of these enzymes in normal cellular and tissue homeostasis or function particularly in mammals (Rhee and Bae, 1997; Assmann and Shimazaki, 1999; Williams, 1999; Liscovitch et al., 2000; Murakami et al., 2015) but, the more recent data reported for plant sPLA_2_s are rather scarce. Therefore, this review focuses on recently acquired information on all sPLA_2_ from plants reported until now with emphasis in *Gm*sPLA_2_s identified in *G. max* (soybean), comparing them with the more relevant published data for several sPLA_2_s obtained from different sources. A comparative description with respect to the sequence characterization, biochemical, molecular, and functional aspects of sPLA_2_s enzymes was done.

## Secretory Phospholipases A_2_ in Plants

In comparison with the animal sPLA_2_, the knowledge generated for sPLA_2_ from plants is still limited, even though when recombinant enzymes from plants have been recently expressed in *Escherichia coli* and yeast and characterized. Some studies about enzyme activities have been reported in more or less crude preparations (Moreau and Morgan, 1988; Mukherjee, 1990; Minchiotti et al., 2008; Murakami et al., 2011).

The first sPLA_2_ purified to homogeneity, sequenced and characterized from plants, was the sPLA_2_ from elm seed endosperm (*Ulmus glabra*) in 1998 (Stahl et al., 1998). Later in 1999, two cDNAs encoding sPLA_2_ (sPLA_2_-I and-II) were isolated from shoots of rice (*Oryza sativa*) and characterized (Stahl et al., 1999). cDNAs full sequences coding for putative sPLA_2_s were obtained from flowers of carnation (*Dianthus caryophyllus*) (Kim et al., 1999). These later clones from carnation and rice have not been further characterized to demonstrate that they encode functional enzymes. With progress in genome sequencing projects, more sPLA_2_s have been identified: in tomato (Lee et al., 2005) and outbreaks of castor bean (*Ricinus communis*) (Domingues et al., 2007). Four isoforms of sPLA_2_ from *Arabidopsis thaliana* have been also isolated, called *At*sPLA_2_-α, -β, -γ, and -δ (Bahn et al., 2003; Lee et al., 2003, 2005; Mansfeld and Ulbrich-Hofmann, 2007; Seo et al., 2008), which have been expressed (Ryu et al., 2005; Mansfeld and Ulbrich-Hofmann, 2007) two isoforms have been studied in tobacco (*Nicotiana tabacum*) (Dhondt et al., 2000; Fujikawa et al., 2005, 2011) and orange (*Citrus sinensis*) (Liao and Burns, 2010). Three cDNA from durum wheat (*Triticum durum*) were isolated and two of them studied in detail (Verlotta et al., 2013; Verlotta and Trono, 2014). A novel sPLA_2_ from opium (*Papaver somniferum*) was purified and characterized (Jablonicka et al., 2016) and two sPLA_2_ from flax (*Linum usitatissimum*) were studied in detail (Gupta and Dash, 2017; Gupta et al., 2017). Moreover, one gene was reported for tomato (*Lycopersicon esculentum*) (Lee et al., 2005) and one gene for maize (*Zea mays*) found in UniProt and mentioned in (Mariani et al., 2012). From our laboratory, five *G. max* phospholipases A_2_ were reported (Mariani et al., 2012), and two of them (*Gm*sPLA_2_-XIA-I and -XIB-II) were cloned, expressed in *E. coli*, further purified from inclusion bodies and the activity was evaluated using organized lipid systems such as mixed micelles and monomolecular films as substrates (Mariani et al., 2015b).

Table 2 summaries the different enzymes found in plants, their origin and source, GenBank accession numbers and the subgroup at which they belong to within the XI group of the PLA_2_ superfamily.

**TABLE 2:** sPLA_2_s from plants, accession numbers, N-terminus characteristics and purification/recombinant process applied.

**Source**	**Name**	**Purification**	**N-terminal^a^**	**Accession number**	**Group XI**	**References**
*A. thaliana*	*At*sPLA_2_-α	cDNA	Recombinant	At2g06925	B	Mansfeld and Ulbrich-Hofmann, 2007
	*At*sPLA_2_-β	cDNA	Mature	At2g19690	A	Lee et al., 2003
(arabidopsis)	*At*sPLA_2_-γ	cDNA	Mature	At4g29460	A	Bahn et al., 2003
	*At*sPLA_2_-δ	cDNA	NR	At4g29470	A	Bahn et al., 2003; Ryu et al., 2005
*R. communis*	*Rc*sPLA_2_α	cDNA	Recombinant	XM002523613	B^b^	Bayon et al., 2015
(castor bean)	*Rc*sPLA_2_β	cDNA	Recombinant	XM002514118	B^b^	Bayon et al., 2015
*C. sinensis*	*Cs*sPLA_2_α	cDNA	Recombinant	GU075396	B^b^	Liao and Burns, 2010
(orange)	*Cs*sPLA_2_β	cDNA	Recombinant	GU075398	A^b^	Liao and Burns, 2010
*D. caryophillus* (carnation)	*Dc*sPLA_2_	cDNA	NR	AF064732	B	Kim et al., 1999
*U. glabra*^c^ (elm)	*Ug*sPLA_2_	Seeds	Purified	NR	NR	Stahl et al., 1998
*G. max*	*Gm*sPLA_2_-XIA-I	cDNA	Mature	BT092274	A	Mariani et al., 2012
(soybean)	*Gm*sPLA_2_-XIA-II	NR	NR	BT094641	A	Mariani et al., 2012
	*Gm*sPLA_2_-XIB-I	NR	NR	BT095220	B	Mariani et al., 2012
	*Gm*sPLA_2_-XIB-II	cDNA	Mature	BT091171	B	Mariani et al., 2015b
	*Gm*sPLA_2_-XIB-III	NR	NR	BT099163	B	Mariani et al., 2012
*L. usitatissimum*	*Lu*sPLA_2_-I	cDNA	Fusion Protein	KU361324	B	Gupta and Dash, 2017; Gupta et al., 2017
(flax)	*Lu*sPLA_2_-II	cDNA	Fusion Protein	KU361325	A	Gupta and Dash, 2017; Gupta et al., 2017
*P. somniferum* (opium)	*Ps*sPLA_2_	cDNA	Recombinant	KU900749	B	Jablonicka et al., 2016
*O. sativa*	*Os*sPLA_2_-I	Seeds	PPfE	AJ238116	A	Lee et al., 2005
(rice)	*Os*sPLA_2_-II	cDNA	Mature	AJ238117	B	Stahl et al., 1999; Guy et al., 2009
	*Os*sPLA_2_-III	NR	NR	AAK50122	B	Lee et al., 2005
*N. tabacum*	*Nt*1PLA_2_	cDNA	Recombinant	AB190177	A	Fujikawa et al., 2011
(tobacco)	*Nt*2PLA_2_	Extract	PPfE	AB190178	B	
*L. esculentum* (tomato)	*Le*sPLA_2_	NR	NR	AI487873	B	Lee et al., 2005; Verlotta et al., 2013
*T. durum*	*Td*sPLA_2_I	cDNA/LE	PPfE	JX021445	A	Verlotta et al., 2013
(durum wheat)	*Td*sPLA_2_II	cDNA/LE	PPfE	JX021446	B	Verlotta et al., 2013
	*Td*sPLA_2_III	cDNA	Recombinant (6× His-TdsPLA_2_III)	JX021447	B	Verlotta et al., 2013; Verlotta and Trono, 2014
	*Td*sPLA_2_IV	cDNA/LE	PPfE	JX021448	B	Verlotta et al., 2013
*Z. mays* (maize)	*Zm*sPLA_2_^d^	NR	NR	EU968759	B	Mariani et al., 2012

*^*a*^Mature, without extra amino acids at the N-terminus after heterologous expression. PPfE, when the enzyme was Partially Purified from Extracted from a plant organ (partial purification, less than 90% purity). LE, leaves extract. Recombinant is indicated when, according to the reported data, it is not known if the expression assayed is in *mature form* or contain *any tags in the final purified recombinant form* (no clearly indicated in the original paper). ^*b*^Classified in this review from proper alignment of the reported sequences. NR, not reported. ^*c*^For sPLA_2_ from *Ulmus glabra* (elm) it was assigned as *Ug*sPLA_2_ since in the original describing paper (Stahl et al., 1998) was named as sPLA_2_ without initial letters of identification. ^*d*^Named as *Zm*sPLA_2_ in this review.*

## Recombinant vs. Native sPla_2_s Proteins: Role of the Intact N-Terminal Preservation

Usually the N-terminus region of sPLA_2_ has an alpha helix domain which forms one wall of the channel through which the hydrophobic substrate entries as reported for groups I and II sPLA_2_s enzymes (according to Dennis, 1973b). Thus, in the case of pancreatic enzyme (group I), when the zymogen is converted into the active form by removing a short portion of the N-terminus, the remaining N-terminal helix is now able to be involved in the binding interfacial membrane (Scott et al., 1990). This would be affected by the extension of seven amino acids at the N-terminus in the zymogen (pro-enzyme) preventing the binding to lipid interfaces. Crystallographic evidence suggests that the zymogen has a more flexible N-terminus compared to the mature protein (van Deenen, 1971).

The effect of an extra amino acid on the N-terminus of pancreatic sPLA_2_ can be critical, for example, if it is of hydrophobic nature (van Scharrenburg et al., 1984). This was observed in the pioneering work of deHaas group, showing that the extension of an amino acid (doubling of the terminal ALA of the mature form) caused a decrease in enzyme catalysis to phosphatidylcholine (PC) short chain substrate presented as micelles or when the substrate was arranged as a lipid monolayer (Slotboom et al., 1977). Furthermore, in the case of porcine pancreatic enzyme, an absolute free amino terminal is required (Dijkstra et al., 1984).

In a recent work with a sPLA2 from group II of *Crotalus atrox* venom, the importance of a native N-terminus was also evident. By using chemically modified enzyme the authors concluded that N-terminal region plays a mechanistic role in catalysis and acts as a surface-active component of the complex interfacial catalytic site (Randolph and Heinrikson, 1982). This structural requirement is also found in other sPLA_2_ expressed in bacteria, such as human sPLA_2_ from synovial fluid (Marki and Hanulak, 1993). It was observed that, when expressing a sPLA_2_ in *E. coli*, the initial MET is not removed from the protein that had an ASN at position 1 in the sequence. This is because the bacterial aminopeptidase does not catalyze the removal of the initial MET if it is followed by ASN. The lipolytic activity of this protein was very low relative compared with the expressed correct N-terminus mature form (Othman et al., 1996). Similarly, another study reported that the protein with an extra MET at its N-terminus had the same pH optimum and prefered substrate compared to the one with native end (without MET), but the activity was drastically reduced (Marki and Hanulak, 1993). Bacterial aminopeptidases remove initial MET efficiently when the amino acid in position 2 of the mature sequence is little and without charge (such as ALA, GLY, SER), but fail when the residue is voluminous and charged as ASN (Hirel et al., 1989). Othman et al. (1996) have substituted the ASN by ALA to express the recombinant protein thus allowing the removal of the initial MET by the bacteria and avoiding a subsequent step of chemical or enzymatic cleavage.

A similar observation was made in sPLA_2_ mutants from Taiwan cobra (Anderson and Dufton, 1997). The addition of a MET at the N-terminus generates structural distortions, and it was postulated that affects the active site through hydrogen bonds network. Moreover, an extra MET decreases the activity with respect to the enzymes with native end (Chiou et al., 2008). Some reports suggest that the N-terminal helix of groups I and II sPLA_2_s acts as a regulatory domain that mediate the interfacial activation (Qin et al., 2005).

The correct design of the heterologous expression of the cloned enzyme is crucial because the recombinant protein must be generated with the correct native N-terminus, without any additional amino acid extension, since any modification or extension of the N-terminus in sPLA_2_ can severely alter the catalytic properties (van Scharrenburg et al., 1984; Othman et al., 1996). This is also valid for any additional N-terminal tag (such as HIS-Tag, frequently used in molecular biology protocols to express recombinant proteins). Both facts make the recombinant protein act as a zymogen like pre-protein.

**FIGURE 1:**
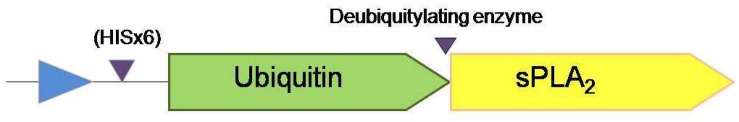
Schematic representation of the system applied for *Gm*sPLA_2_s expression and purification. Triangles indicate the position where is the polyHIS-tag motif and the position where the deubiquitylating enzyme cut in order to release the protein with its native N-terminus without any extension.

In this sense, the sPLA_2_s obtained from *G. max* were expressed without N-terminal extension (Mariani et al., 2012, 2015b) by using the pHUE vector system that utilizes the ubiquitin fusion technique (Catanzariti et al., 2004), which allows easy purification and high yield of recombinant proteins (see Figure 1). The *E. coli* pHUE vector permits the expression of a particular protein as HIS-tagged ubiquitin fusion. Then, the HIS-tag-ubiquitin-sPLA_2_ fusion is further processed by the deubiquitylating enzyme used to cleave off the fusion to obtain the protein of interest free of any N-terminal extension (Figure 1).

In the particular case of the mature protein *Gm*sPLA_2_-XIB-II, the LEU amino acid at the N-terminus was mutated to an ALA, to optimize the chance of obtaining the correct refolding as previously recommended (Kohler et al., 2006). In *At*sPLA_2_-α, it was shown that an uncleaved signal peptide of the pre-processed forms produced a significant suppression of activity compared with the corresponding mature protein form (Ryu et al., 2005). Moreover, other sPLA_2_s from plants were expressed without the signal peptide (Mansfeld et al., 2006; Guy et al., 2009). In animals, a correct and functional sPLA_2_ from *Bothrops diporus* was produced without any extra extension at the N-terminus (Yunes Quartino et al., 2012). Using the ubiquitin/deubiquitinase system, in the latter case, it was clearly shown that the recombinant protein had the same interfacial catalytic profile when compared to the native one (Yunes Quartino et al., 2015).

As sPLA_2_ activity is very sensitive to N-terminus modifications, in Table 2 we include all sPLA_2_s from plants known until today and the process originally reported to obtain the final protein (either proteins purified from plant extracts, in a mature recombinant form or with an additional tag). It should be noted that not all reported information disclosed the sequence of phospholipase A_2_ either cloned or purified.

However, similar to its counterpart in animals, sPLA_2_s from plants have N-terminal signal peptides that were predicted to direct protein secretion into the extracellular or intracellular space (Bahn et al., 2003; Lee et al., 2003). It is noteworthy that some sPLA_2_s from plants have the sequences KTEL, KFEL, and KLEL at the C-terminal which are similar to the endoplasmic reticulum retention sequences KDEL and HDEL reported for animals (Matsushima et al., 2003). Even when this putative KxEL endoplasmic reticulum (ER) retention sequence (Pagny et al., 2000; Seo et al., 2008) is present in some plant sPLA_2_, the biochemical significance is still unknown (see Supplementary Figure S1).

## *Gm*sPla_2_s Gene Family, Classification, and Domain Structure

In *G. max*, five sPLA_2_s isoforms were identified (Mariani et al., 2012), named as *Gm*sPLA_2_-XIA-I, *Gm*sPLA_2_-XIA-II, *Gm*sPLA_2_-XIB-I, *Gm*sPLA_2_-XB-II, and *Gm*sPLA_2_-XIB-III. Detailed information about the genes and proteins are shown in Table 3. As indicated above, the extension of the N-terminus of the mature protein is crucial for the activity, we show in Supplementary Figure S5 all the sequences of the sPLA_2_s of known plants with their signal sequence and their point of theoretical cut using the programs available online.

**TABLE 3:** Sequence characteristics of the *Gm*sPLA_2_s^a^.

**Name**	**Full-length cDNA (nt)**	**Open reading frame (ORF) (nt)**	**Residues of native protein with signal peptide**	**Residues of mature protein**
*Gm*sPLA_2_-XIA-I	789	417	138	114
*Gm*sPLA_2_-XIA-II	875	417	138	115
*Gm*sPLA_2_-XIB-I	826	474	157	128
*Gm*sPLA_2_-XIB-II	762	471	156	128
*Gm*sPLA_2_-XIB-III	821	477	158	128

*^*a*^In Supplementary Figure S5 it is shown the complete sequence with the N-terminal region with the signal peptide and the putative theoretical site of cleavage for all sPLA_2_ reported for plants. Signal peptides for each sequence was determined by using the signalP 3.0 server (http://www.cbs.dtu.dk/services/SignalP-3.0/).*

Moreover, the genes encoding for *Gm*sPLA_2_-XIA-I and *Gm*sPLA_2_-XIB-I are located in chromosome I, *Gm*sPLA_2_-XIA-II y *Gm*sPLA_2_-XIB-II are positioned in chromosome 7 and the gene of *Gm*sPLA_2_-XIB-III is located in chromosome 8. Whereas *Gm*sPLA_2_-XIB-I, *Gm*sPLA_2_-XIB-II, and *Gm*sPLA_2_-XIB-III possess three introns and four exons, the genes of *Gm*sPLA_2_-XIA-I and *Gm*sPLA_2_-XIA-II have two introns and three exons, respectively (see Supplementary Material in Mariani et al., 2012). These facts are indicative that during the course of evolution events of divergence and duplication might have occurred as it was suggested previously for *At*sPLA_2_s (Lee et al., 2005).

**FIGURE 2:**
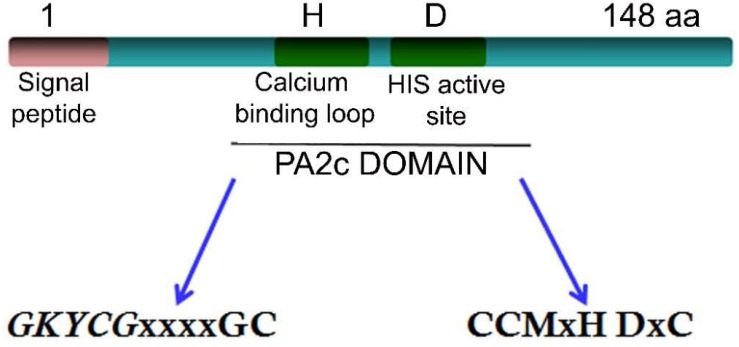
Schematic representation of the *Gm*sPLA_2_ gene.

All sPLA_2_s sequences found in plants hold a PA2c (SMART accession number SM00085^1^) domain that contains the highly conserved Ca^2+^-binding loop (YGKYCGxxxxGC) (see Figure 2). The active site motif (DACCxxHDxC) that holds the highly conserved HIS/ASP pair (Laigle et al., 1973) corresponds to position 49/50 for *Gm*sPLA_2_-XIA-I, 47/48 for *Gm*sPLA_2_-XIA-II and 62/63 for *Gm*sPLA_2_-XIBs whereas for *At*sPLA_2_α and *At*sPLA_2_γ, it corresponds to the position 62/63 and 7/48, respectively (Mansfeld et al., 2006).

**FIGURE 3:**
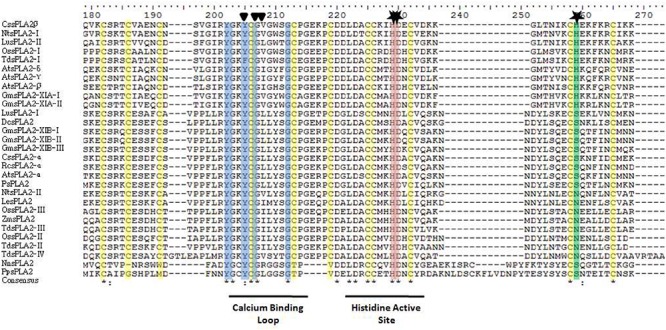
Sequence alignment of the sPLA_2_s reported from plants: analysis of the deduced amino acid sequences. The alignment was performed by using the Clustal X v2.1 program (http://www.clustal.org) and edited with jalview program. The triangles denote the amino acid residues involved in binding Ca^2+^ and stars denote amino acid residues putatively involved in catalysis. In yellow, CYS residues are marked. For abbreviation code of plants sPLA_2_ see Table 2. Two animals sPLA_2_ are included at the bottom of the figure for comparison: *Nn*sPLA_2_ from *Naja naja* (cobra) venom and *Pp*sPLA_2_ from pig pancreatic juice (*Sus scrofa*). The accession numbers of sPLA_2_ indicated in this figure are provided as Supplementary Appendix S1.

However, there is a dissimilarity that remains unclear in the HIS/ASP of the catalytic dyad in sPLA_2_s from plants compared with those found in animals (Mansfeld et al., 2006). It was proposed that water molecules assist in the Ca^2+^ coordination at the HIS48-ASP49 active site in bovine pancreatic *bp*sPLA_2_ (Bahnson, 2005), the roles of ASP99 in this sPLA_2_ (Kumar et al., 1994), and ASP64 in bee venom sPLA_2_ (West et al., 2013) were also claimed to take part in the hydroxyl-imidazole-carboxylate motif (Annand et al., 1996). However, for sPLA_2_ plant enzymes this important catalytic residue is replaced by an HIS or an ASN residue in enzymes from group XIA and by a SER or an ASN in those enzymes belonging to group XIB (Mansfeld et al., 2006) as shown in the alignment in Figure 3. Mansfeld et al. (2006) demonstrated that SER, ASN, or HIS in plant sPLA_2_s may fulfill the catalytic role assigned to ASP in animal’s sPLA_2_s (Mansfeld et al., 2006). Sequence alignment also reveals that, contrary to *Os*sPLA_2_ isoforms, the ASP residue of the highly conserved HIS/ASP catalytic dyad of the animal counterpart is replaced by an HIS residue in the durum wheat *Td*sPLA_2_ isoform I, and by an ASN residue in all of the others durum wheat sPLA_2_ isoforms (Verlotta et al., 2013), see Figure 3 for more details of others sPLA_2_ from plants. Even though the comparison showed low homology among them within the overall amino acid sequences, both the catalytic site and the Ca^2+^ binding loop are highly conserved (Figure 3). Other relevant conserved residues within the Ca^2+^ binding loop are the two TYR and two GLY residues which are involved in the hydrogen bonding network reported for both animal and plant sPLA_2_s (Lee et al., 2005). A more perusal view of this domain offers additional information. The more conserved domain YGKYCG seems not to be exclusive, a change in the second TYR residue was observed for *Td*sPLA_2_-I changing to YGKFCG. Also, the following hydrophobic domain mainly formed by the LL pair may be VL, IL, IM, VS, IG, or VG (see Figure 3). However, the putative role of these differences on calcium affinity or phospholipase activity was not elucidate yet.

The mature proteins of both groups XIA and XIB contain 12 CYS residues (Figure 3) known to form six structural disulfide bonds that also are present in the same position as other known sPLA_2_s from plants (Mansfeld et al., 2006). CYS residues are essential for secreted sPLA_2_s and it has been shown to play a relevant role in the structural stability in mature sPLA_2_s (Six and Dennis, 2000; Mariani et al., 2015b).

The HIS residue (at position 49 in *Gm*sPLA_2_-XIA-I, 47 in *Gm*sPLA_2_-XIA-II and at 62 in *Gm*sPLA_2_-XIB-II, -II, and -III) was suggested to play a crucial role in the nucleophilic attack at the *sn-2* bond in the glycerol backbone of phospholipids for all sPLA_2_s (Six and Dennis, 2000; Berg et al., 2001; Burke and Dennis, 2009).

All plants sPLA_2_s are low MW enzymes (12–18 kDa) with the exception of *Cs*sPLA_2_β from Citrus that has an unexpected high MW (Table 4). The theoretical isoelectric points (*pI*) for each sPLA_2_ are shown also in Table 4. As it can be observed, four of the putative *Gm*sPLA_2_s are rather acidic or neutral (*Gm*sPLA_2_-XIA-I, *Gm*sPLA_2_-XIB-I, -II, and -III) as reported for sPLA_2_s isolated from *Bothrops diporus* venom (de Haas et al., 1968; Daniele et al., 1997). Acidic sPLA_2_s were also reported for some enzymes found in the Crotalinae subfamily (dos Santos et al., 2011) and those found in rice (isoforms I and III) (Lee et al., 2005). On the other hand, the expected *pI* of *Gm*sPLA_2_-XIA-II is slightly alkaline similar to those of all the sPLA_2_s found in *Arabidopsis* (Lee et al., 2005) and *Papaver somniferum* (Jablonicka et al., 2016); whereas other sPLA_2_s have a *pI* almost neutral as those found for carnation and tomato (Lee et al., 2005).

**TABLE 4:** Molecular weight, isoelectric point, and specific activity of different sPLA_2_s from plants.

**Origin**	**Name**	**Mature protein MW (kDa)**	***pI***	**Reported activity (μmol min^–1^ mg^–1^ protein) and substrate**	**References**
*A. thaliana*	*At*sPLA_2_-α	14.2	7.7	16.7 (DOPC)	Lee et al., 2005; Mansfeld and Ulbrich-Hofmann, 2007
	*At*sPLA_2_-β	16.3	8.2	0.53 (PC)	Lee et al., 2003, 2005
(arabidopsis)	AtsPLA_2_-γ	17.5	8.3	NR	Bahn et al., 2003; Lee et al., 2005
	AtsPLA_2_-δ	18.0	7.7	NR	Lee et al., 2005
*D. caryophillus* (carnation)	*Dc*sPLA_2_	12.4	6.9	NR	Lee et al., 2005
*R. communis* (castor bean)	*Rc*sPLA_2_α	14	6.3^a^	52.3 pmol min^–1^ mg^–1^ ([^14^C]18:1-PC)	Bayon et al., 2015
*C. sinensis* (orange)	*Cs*sPLA_2_α	17.1	6.9^a^	0.013^d^ arachidonoyl Thio-PC	Domingues et al., 2007; Liao and Burns, 2010
	*Cs*sPLA_2_β	31.6	8.1^a^	0.013^d^ arachidonoyl Thio-PC	Liao and Burns, 2010
*U. glabra* (elm)	*Ug*sPLA_2_	13.9	NR	90 (PCPC)	Stahl et al., 1998; Lee et al., 2005
*G. max*	*Gm*sPLA_2_-XIA-I	12.3	6.9	0.44 (DLPC)	Mariani et al., 2012, 2015b
(soybean)	*Gm*sPLA_2_-XIA-II	12.6	7.4	NR	Mariani et al., 2012, 2015b
	*Gm*sPLA_2_-XIB-I	13.9	5.7	NR	Mariani et al., 2012, 2015b
	*Gm*sPLA_2_-XIB-II	13.9	5.7	0.25 (DLPC)	Mariani et al., 2012, 2015b
	*Gm*sPLA_2_-XIB-III	14	6.8	NR	Mariani et al., 2012, 2015b
*L. usitatissimum*	*Lu*sPLA_2_-I	17.9	6.7	∼2 (PC_LIN_)	Gupta and Dash, 2017
(flax)	*Lu*sPLA_2_-II	15.7	8.8	∼2.7 (PC_LIN_)	Gupta and Dash, 2017
*P. somniferum* (opium)	*Ps*sPLA_2_	14	6.9	∼7 (DOPC)	Jablonicka et al., 2016
*O. sativa* (rice)	*Os*sPLA_2_-I^b^	12.9	7.9	145 (*sn1*-palmitoyl-*sn2*-[^14^C]caproyl-PC)	Stahl et al., 1999; Lee et al., 2005
	*Os*sPLA_2_-II^b^	13.8	5.5	145 (*sn1*-palmitoyl-*sn2*-[^14^C]caproyl-PC)	Stahl et al., 1999; Lee et al., 2005; Guy et al., 2009
	*Os*sPLA_2_-III	13.5	4.8	NR	Lee et al., 2005
*N. tabacum*	*Nt*1PLA_2_	17.0	8.57	1.2 (POPC)	Fujikawa et al., 2005
(tobacco)	*Nt*2PLA_2_	12.7	6.8^a^	NR	Lee et al., 2005
*L. esculentum* (tomato)	*Le*sPLA_2_	13.9	6.9	NR	Lee et al., 2005
*T. durum*	*Td*sPLA_2_s-I	∼14^c^		1.55^d^ (PC_LIN_)	Verlotta et al., 2013
(durum wheat)	*Td*sPLA_2_s-II	∼15.7^c^		1.55^d^ (PC_LIN_)	Verlotta et al., 2013
	*Td*sPLA_2_s-III	∼13.9	4.5^a^	3.2 (PC_LIN_)	Verlotta and Trono, 2014
	*Td*sPLA_2_s-IV	∼17^c^		1.55^d^ (PC_LIN_)	Verlotta et al., 2013
*Z. mays* (maize)	*Zm*sPLA_2_	14.3^a^	5.43^a^	NR	This review

*^*a*^Indicates the *pI* or MW calculated by using the on line interface https://web.expasy.org/compute_pi/. ^*b*^The mixed enzyme activity was determined from an 8-day-old rice shoots extract (Stahl et al., 1999). ^*c*^Corresponds to full length sequence (Verlotta et al., 2013). ^*d*^The enzyme activity of all isoforms (per gram of dry extract) was determined from a direct orange (Liao and Burns, 2010) and wheat (Verlotta et al., 2013) extract. NR, not reported.*

The functional role of the diverse *pI*s found in different sPLA_2_s has not clearly been elucidated yet.

Another relevant domain information is that the enzymes from the different subgroups differ in the third Ca^2+^ coordinating amino acid, being a GLY residue in subgroup XIA or LEU residue in subgroup XIB (see Figure 3). The ASP located upstream in the sequence of the common HIS/ASP catalytic dyad found in animal sPLA_2_s does not correlate in the counterpart found in plants. Instead of this additional ASP residue, the plant enzymes that belong to group XIA contain an HIS residue, and the enzymes belonging to group XIB contain either a SER or an ASP residue (Mansfeld et al., 2006). The functional role of these differences with regard to the catalytic properties has not been completely elucidated yet.

## *Gm*sPla_2_s Classification in the sPla_2_ Superfamily

Secretory phospholipases in plants superfamily is composed of multiple members represented by multiple isoforms distinguishable by their structural, catalytic and physiological characteristics. sPLA_2_ are within the most populated group of PLA_2_ in nature which in turn is classified into 15 subgroups (Six and Dennis, 2000). In this context, the plant sPLA_2_s were classified into a separate group (group XI) (Meneghetti and Maggio, 2013), which, in turn, could be subdivided into two categories named XIA and XIB because of differences in MW and deviating sequences in the N- and C-terminal regions of the mature enzyme (Six and Dennis, 2000).

**FIGURE 4:**
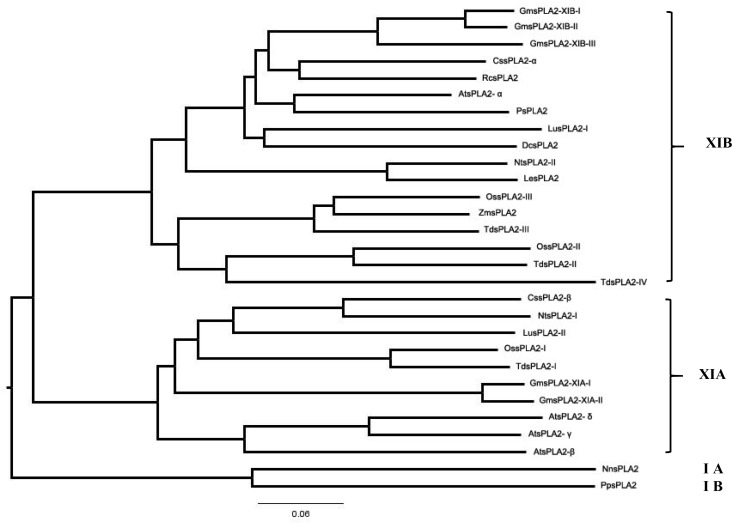
Phylogenetic tree of the deduced amino acid sequences of all reported plant sPLA_2_s. The program used to perform the sequence alignment was Clustal X (version 2.0) and to produce phylogenetic analysis the phylogenetic tree generation methods from the ClustalW2 package was used (on line tool http://www.ebi.ac.uk/Tools/phylogeny/clustalw2_phylogeny/). To draw the tree we used the Figtree v.1.4.4 program (http://tree.bio.ed.ac.uk/software/figtree/). The accession numbers of sPLA_2_ indicated in this figure are provided as Supplementary Appendix S1.

Figure 4 shows the phylogenetic classification into the two subgroups of all the sPLA_2_s from plants known until now. This way, *Gm*sPLA_2_-XIA-I and -II are taking part of group XIA, which includes *At*sPLA_2_-γ, *At*sPLA_2_-β, *At*sPLA_2_-δ, *O. sativa* isoform I, *N. tabacum* isoform I, *T. durum* isoform I, *C. sinensis* isoform β, and *L. usitatissimum* isoform II. Whereas two of the enzymes of *G. max* correspond to the subgroup XIA, three are grouped in the subgroup XIB (Mariani et al., 2012) named as *Gm*sPLA_2_-XIB-I, -II, and -III together with *At*sPLA_2_-*α*, *O. sativa*-II, -III, and -IV, *D. caryophillus*, *N. tabacum* isoform II, *Z. maize*, *R. communis* isoform α*, P. somniferum*, *T. durum*-II, -III, and -IV, *L. esculentum*, *C. sinensis* isoform α, and *L. usitatissimum* isoform I.

The data show a close evolutionary relationship among all sPLA_2_s from plants (see Figure 4). The highest level of similarity in amino acid sequences was observed between *Gm*sPLA_2_-XIA-I and *Gm*sPLA_2_-XIA-II, being of 95.5%, whereas between *Gm*sPLA_2_-XIB-I and *Gm*sPLA_2_-XIB-II the level of similarity is of 94.5% (Mariani et al., 2012) (see Supplementary Figure S2). Moreover, between *Le*sPLA_2_ and *Nt*sPLA_2_-II the level of similarity is of 90.4% and between *Td*sPLA_2_-I and *Os*sPLA_2_-I, *Gm*sPLA_2_-II and *Gm*sPLA_2_-III, *P*sPLA_2_ and *Rc*sPLA_2_-α and *At*sPLA_2_-δ and *At*sPLA_2_-γ the levels of similarity are of 89.9, 87.3, 83.7, and 82.7%, respectively.

## Tridimensional Structure

Although the sPLA_2_ sequences from different sources differ significantly, the tridimensional structures have many features in common. There are more than 40 sPLA_2_s entries in the Protein Data Bank (PDB^2^) from all sources. Native and complex structures of sPLA_2_s simulated with mimic substrate have helped to identify the catalytically important residues involved in the active site (Pan et al., 2002).

The tridimensional structure of many sPLA_2_s, such as porcine pancreas or bee venom (Dijkstra et al., 1984; Scott et al., 1990), has been elucidated by X-ray crystallography which revealed a common, rigid and highly conserved region with a similar tridimensional architecture compared with those from plants. The active site is not directly accessible to the aqueous phase and is within a rather local hydrophobic environment denoted as “*i-face*” that allows the interaction with the substrate in its monomeric form (Dijkstra et al., 1981). The putative residues involved in the “*i-face*” of some sPLA_2_ from plants are shown in Table 5.

**TABLE 5:** Reported and proposed amino acid residues involved in the “*i-face*” of several sPLA_2_.

**sPLA_2_ name**	**Proposed amino acids in the *i-face***	**References**
*Pp*sPLA_2_ (Group IB)	L^2^, W^3^, R^6^, L^19^, M^20^, L^31^, and Y^69^	Kuipers et al., 1991
*Bp*sPLA_2_	L^2^, W^3^, F^5^, I^9^, F^22^, L^31, 63–65^, and Y^69^	Yu et al., 1999b
*Gm*sPLA_2_-XIA-I	V^18^, G^19^, V^28^, H^49^, H^64^, L^101^, A^102^, I^103^, L^104^, L^105^, and L^108^	Mariani et al., 2012
*Gm*sPLA_2_-XIB-II	F^25^, S^27^, L^31^, V^112^, A^116^, L^119^, V^123^, L^124^, and P^127^	Obtained by using the on-line platform OPM (Lomize et al., 2012)
*Os*sPLA_2_-II	A^29^, P^30^, V^65^, Y^72^, and L^41^	Guy et al., 2009

**Pp*, *P. pancreas*; *Bp*, *B. pancreas*; *Gm*, *G. max*; *Os*, *O. sativa*.*

One of the first sPLA_2_ “*i-face*” identified was for the secreted pig pancreatic enzyme (Bai et al., 2008). In *Gm*sPLA_2_-XIA-I the residues found in the putative “*i-face*” are VAL18, GLY19, VAL28, HIS49, HIS64, LEU101, ALA102, ILE103, LEU104, LEU105, and LEU108. Table 5 shows the putative amino acids proposed to be in contact with the membrane for different sPLA_2_s enzymes.

The binding of sPLA_2_ to the membrane is energetically favorable (Table 6) and, keeping in mind that most of the residues situated in the *i-face* are hydrophobic, the overall domain constitutes a hydrophobic environment that surrounds the active catalytic site. Hydrophobic side chains of the residues forming the “*i-face*” would be able to partition to the hydrophobic core, which allows the anchoring of the enzyme to the membrane, excluding water molecules in the region surrounding the active site and the diffusion of the substrate to the pocket of the active site to be hydrolyzed. The general molecular conformation proposed for plants sPLA_2_ is in agreement with the general vision proposed for secreted phospholipases of animal source (Scott et al., 1990).

**TABLE 6:** Orientation of different sPLA_2_s at the membrane interface.

**Protein**	**Depth/hydrophobic thickness**	**ΔG_transfer_ (kcal/mol)**	**Tilt angle**	**Embedded residues**
*Gm*sPLA_2_-XIB-II^a^	2.8 ± 0.9 Å	−10.4	71 ± 4°	F^25^, S^27^, L^31^, V^112^, A^116^, L^119^, V^123^, L^124^, and P^127^
*Gm*sPLA_2_-XIA-I^b^	1.0 ± 2.8 Å	−1.0	69 ± 20°	P^114^
*Os*sPLA_2_-II^c^	4.1 ± 0.5 Å	−10.6	71 ± 2°	G^3^, L^6^, A^25^, L^28^, Y^30^, G^31^, I^116^, R^120^, and D^121^
*Lu*sPLA_2_I^d^	4.1 ± 0.6 Å	−7.1	86 ± 3°	F^27^, A^29^, V^30^, P^32^, and L^33^
*Lu*sPLA_2_II^d^	1.9 ± 1.2 Å	−5.5	86 ± 26°	F^24^ and L^102^
*Naja sagittifera*^e^ *Ns*sPLA_2_	1.6 ± 0.4 Å	−4.3	85 ± 2°	D^20^ and K^65^
*Naja naja*^f^ *Ns*sPLA_2_	3.6 ± 0.3 Å	−6.7	86 ± 2°	Y^3^, W^19^, W^61^, and F^64^
*Pig pancreatic*^g^ *Pp*sPLA_2_	2.4 ± 2.5 Å	−1.9	32 ± 16°	L^64^

*^*a*^Structure were modeled in a similar way than that described for *Gm*sPLA_2_-XIA-I in Mariani et al. (2012), the PMDB accession number is PM0082160. ^*b*^Structure modeled in Mariani et al. (2012), the PMDB accession number is PM0082161. ^*c*^Obtained from PBD 2WG7. ^*d*^PMDB identified as PM0080416 (*Lu*sPLA_2_-I) and PM0080415 (*Lu*sPLA_2_-II). ^*e*^Crystal structure of *Naja sagittifera* was reported in Jabeen et al. (2005). PDB 1MH8. ^*f*^*Naja naja Nn*sPLA_2_. PDB 1A3D. ^*g*^*Pig pancreatic Pp*sPLA_2_. PDB 1PIR.*

Physically, the soluble sPLA_2_ protein must penetrate the phospholipid interface to exert its action. Therefore, the successful binding surface is located where the substrate is a prerequisite in the catalytic cycle, and this property can determine some specific characteristics of the enzyme activity. However, there are a limited number of charged residues in the flat topography of the “*i-face*” (see Table 5) that could modulate further interaction with the interface of the substrate in a way which has not been fully elucidated yet (Jain and Berg, 2006).

It is important to note that even when the energetic to membrane binding is favorable according to the available on-line calculation program (Lomize et al., 2012) used for some sPLA_2_s, the residues involved in the “*i-face*” differ for the same enzyme if a different approach is used instead (compare Tables 5, 6).

**FIGURE 5:**
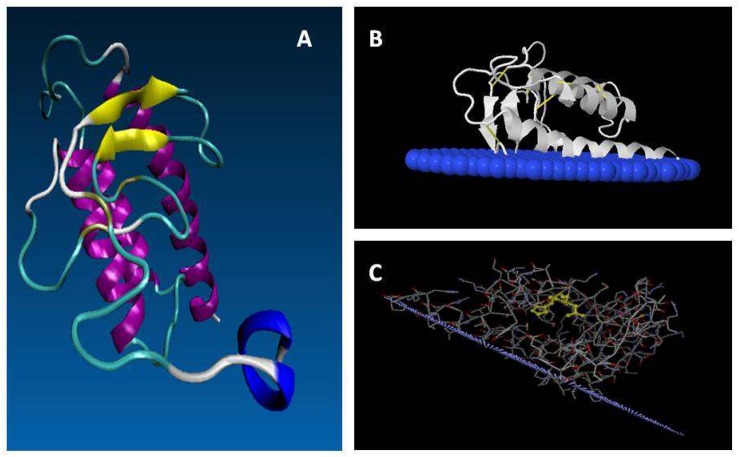
Putative mature structure of GmsPLA_2_-XIA-I. **(A)** Proposed structure from homology modeling of GmsPLA2-XIA-I (Mariani et al., 2012). Yellow: beta sheet strand; magenta: alpha-helix; blue: C-terminal; cyan: turns; white: coils. **(B,C)** Molecular simulation of the interaction between *Gm*sPLA_2_-XIB-II with the membrane interface, simulated with the OPM (Orientation of Proteins in Membrane) database online service (opm.phar.umich.edu/; see Lomize et al., 2012). In panel **(B)** blue: interfacial membrane; white: protein. **(C)** Light purple represents the interfacial membrane; the sticks denote the protein amino acids with the H/D dyad highlighted in yellow.

To date, only few structures corresponding to sPLA_2_s from plants were reported in the PDB or in the Protein Model Database (PMDB^3^) and correspond to *O. sativa* (rice) isoform II (PBD 2WG7), which belongs to the group XIB, and its tertiary structure was recently determined by X-ray crystallography to 2.0 Å resolution (Guy et al., 2009). Moreover, homology modeling and molecular dynamics were used to elucidate the structure of sPLA_2_ isoform α from *Arabidopsis* (Mansfeld et al., 2006) but its PDB is not available. The predicted models of *Lu*sPLA_2_s proteins were elucidated and submitted to PMDB identified as PM0080416 (*Lu*sPLA_2_-I) and PM0080415 (*Lu*sPLA2-II) (Gupta and Dash, 2017). The structure of *Gm*sPLA_2_-XIA-I was modeled by using homology modeling and molecular dynamics (Mariani et al., 2012) and also *Gm*sPLA_2_-XIB-II by using a similar methodology (see Figure 5, modeled structures in PDB format were not uploaded in the PDB). The data corresponding to *Pig pancreatic* (*Sus scrofa*), *Naja naja* (Indian cobra), *Naja sagittifera* (Andaman cobra venom) are also indicated in Table 6 for comparison in order to include sPLA_2_ able to hydrolyze aggregate lipids structured in a high packing organization, as it occurs with sPLA_2_ from cobra venom, or only at low packing as it certainly happens with sPLA_2_ from pig pancreas (see below and Table 10).

The structure of rice sPLA_2_ shows that the half N-terminal chain contains mainly structured loops, including the conserved calcium binding loop domain together with two short anti-parallel β-strands. The half C-terminal is folded into three anti-parallel α-helix, in which two of them are highly conserved among others sPLA_2_s, containing the crucial catalytic HIS residue and the calcium binding/coordinating ASP residues (Guy et al., 2009). This overall general folded conformation seems to be shared by almost all known sPLA_2_ from plants. The complete putative mature structure of *Gm*sPLA_2_-XIA-I protein was reported using homology modeling and molecular dynamics simulations (Mariani et al., 2012). The most mobile regions are the N- and C-terminal, followed by the loops in residues 74–85, 53–62, 34–37 that connect, respectively, the last two helices, the first with the second helix, and the last beta-sheet with the first helix (see Figure 5). As other sPLA_2_s in the family, the dominant secondary structure is the α-helix, with only a small portion of beta sheet with abundant regions containing turns and bends. The observations indicate that the terminal helix is rather a dynamic region and has three principal conformations: one fully helical, other with the last seven residues in coil, and the third one with a kink plus coil (Mariani et al., 2012). As noted before, this behavior can be attributed to a low number of hydrophobic contacts of this region, a high aqueous exposed area and the presence of a highly flexible GLY98 residue (Mariani et al., 2012).

The active site of the sPLA_2_ protein contains a crucial calcium ion cofactor commonly present in other plant sPLA_2_s (Mansfeld et al., 2006; Guy et al., 2009) that is important in the catalytic mechanism and is a requisite for full enzyme activity. The HIS-ASP pair constitutes the active center and the calcium binding loop (see Figure 3) is essential for the proper function of the enzyme (Scott et al., 1990). All sPLA_2_s catalyze the hydrolysis through the same mechanism: an abstraction of a proton from a water molecule followed by a nucleophilic attack on the *sn-2* bond position of the diacylglycerophospholipids (Jorgensen et al., 1983; Berg et al., 2001). NMR structural studies of porcine pancreas sPLA_2_ show that the N-terminus is flexible with no defined structure in solution, unlike what it was evidenced by crystallography. It was hypothesized that this flexibility in solution would be related to the near null activity against monomeric substrate form [more unstructured unbound state (van den Berg et al., 1995)].

## Enzymatic Properties of Plants sPla_2_s

### Optimum Conditions for Plants sPLA_2_s Catalysis

The sPLA_2_s from *N. tabacum* and elm have optimum pH in the range of 8–10 and 8–9, respectively (Stahl et al., 1999; Fujikawa et al., 2005). In *Arabidopsis* the optimum pH ranges for the activities are pH 6–11, 6–7, 7–9, and 8–9 for *At*sPLA2-α, -β, -γ, and -δ, respectively (Lee et al., 2005). Nevertheless, a similar situation was found for almost all the sPLA_2_s found in plants or animals. The pH optimum was at around 7 for *Gm*sPLA_2_-XIA-I and -XIB-II (see Table 7), when using mixed micelles of DLPC:Triton X-100 as substrate in presence of calcium 10 mM. The optimum pH for pancreatic sPLA_2_ was reported to be 8 (de Haas et al., 1968; Fujikawa et al., 2005) similar to that reported for bee venom (Daniele et al., 1997). For human non-pancreatic PLA_2_ optimum pH is in between 8 and 10 (Kramer et al., 1989). However, it should be mentioned that different substrates (including different aggregation presentation of substrate) have been used to determine optimum pH for the different sPLA_2_s reported in the literature.

**TABLE 7:** Optimum requirements deduced for catalytic activity of the different sPLA_2_s found in plants.

**Source**	**Name**	**pH**	Calcium requirement^*^	**T (°C)**	**References**
*A. thaliana*	*At*sPLA_2_-α	6–11	mM	30–40	Lee et al., 2005; Mansfeld et al., 2006; Mansfeld and Ulbrich-Hofmann, 2007
	*At*sPLA_2_-β	6–7	>0.5 mM	30	Lee et al., 2003, 2005
(arabidopsis)	*At*sPLA_2_-γ	7–9	>0.5 mM	30	Bahn et al., 2003; Lee et al., 2005
	*At*sPLA_2_-δ	8–9	>0.5 mM	NR	Lee et al., 2005
*R. communis* (castor bean)	*Rc*sPLA_2_α	8	10 mM	30	Bayon et al., 2015
*C. sinensis*	*Cs*sPLA_2_α	7.4	10 mM	25	Liao and Burns, 2010
(orange)	*Cs*sPLA_2_β	7.4	10 mM	NR	Liao and Burns, 2010
*D. caryophillus* (carnation)	*Dc*sPLA_2_	NR	NR	NR	–
*U. glabra* (elm)	*Elm*sPLA_2_	8–9	10–15 mM	30	Stahl et al., 1998
*G. max* (soybean)	*Gm*sPLA_2_-XIA-I	6–7	>1 mM	40–60	Mariani et al., 2015b
	*Gm*sPLA_2_-XIA-II	NR	NR	NR	–
	*Gm*sPLA_2_-XIB-I	NR	NR	NR	–
	*Gm*sPLA_2_-XIB-II	6–7	>1 mM	40–60	Mariani et al., 2015b
	*Gm*sPLA_2_-XIB-III	NR	NR	NR	–
*L. usitatissimum* (flax)	*Lu*sPLA_2_-I	9	1 mM	NR	Gupta and Dash, 2017
	*Lu*sPLA_2_-II	9	1 mM	NR	Gupta and Dash, 2017
*P. somniferum* (opium)	*P*sPLA_2_	7	NR	37	Jablonicka et al., 2016
*O. sativa* (rice)	*Os*sPLA_2_-I	8	10 mM	30	Stahl et al., 1999; Guy et al., 2009
	*Os*sPLA_2_-II	8	10 mM	30	Stahl et al., 1999; Guy et al., 2009
	*Os*sPLA_2_-III	NR	NR	NR	–
*N. tabacum* (tobacco)	*Nt*1sPLA_2_	8–10	<1 mM	37	Fujikawa et al., 2005, Fujikawa et al., 2011
	*Nt*2sPLA_2_	–	–	–	NR
*L. esculentum* (tomato)	*Le*sPLA_2_	NR	NR	NR	NR
*T. durum*	*Td*sPLA_2_-I	9	>2 mM	25	Verlotta et al., 2013
(durum wheat)	*Td*sPLA_2_-II	9	>2 mM	25	Verlotta et al., 2013
	*Td*sPLA_2_-III	9	1 mM	25	Verlotta et al., 2013; Verlotta and Trono, 2014
	*Td*sPLA_2_-IV	9	>2 mM	25	Verlotta et al., 2013
*Z. mays* (maize)	*Zm*sPLA_2_	–	–	–	NR

^*^For *Arabidopsis* sPLA_2_s-β, -γ, and -δ, a μM requirement was reported without specifying the precise concentration. NR, not reported.

Only few sPLA_2_s were investigated about the optimum temperature and stability. *Gm*sPLA_2_s-XIA-I and -XIB-II demonstrate to be very stable when increasing the temperature (Mariani et al., 2015b) as previously determined by using an sPLA_2_ homogenate (Minchiotti et al., 2008). This proves that these enzymes are highly resistant to temperature denaturation due in part to the disulfide bridges that are postulated to be involved in the stability of sPLA_2_s (Berg et al., 2009; Murakami et al., 2010). Table 7 shows the optimal temperature reported for several sPLA_s_ from plants.

The optimum calcium concentrations for activity of *Gm*sPLA_2_-XIA-I and -XIB-II are in the micromolar range using DLPC:Triton X-100 mixed micelles as substrates (Table 7). This micromolar calcium requirement is rather unusual for sPLA_2_s enzymes that mostly possess millimolar requirement (Six and Dennis, 2000). Moreover, the same behavior was observed for the activities of *At*sPLA_2_-β, -γ, and -δ (Lee et al., 2005). It is important to remark that none of these secreted enzymes (either from animals or plants) exhibit activity in absence of calcium. Particularly, for *At*sPLA_2_-α the activity augmented as the calcium concentration increased up to 10 mM and for elm sPLA_2_ the range of calcium concentration for optimal activity was around 10–15 mM CaCl_2_ (Stahl et al., 1998; Lee et al., 2005; Mansfeld and Ulbrich-Hofmann, 2007). However, to achieve 50% of maximal enzyme activity a concentration of 0.5 mM CaCl_2_ was sufficient, at least, for these two latter enzymes. The maximal activity for sPLA_2_ from *N. tabacum* was detected above 1 mM CaCl_2_. This behavior is similar to that observed for the most animal sPLA_2_s, which require millimolar concentrations of Ca^2+^ and have no activity in the absence of this cation (Six and Dennis, 2000; Fujikawa et al., 2005). Even when it is evident the molecular differences among the enzymes in the sPLA_2_ family, the absolute requirement of Ca^2+^ for hydrolysis is indicative that all of them share a common mechanism for lipid hydrolysis. For durum wheat sPLA_2_ the activity continuously increased as Ca^2+^ concentration increased with a plateau close to 2–4 mM CaCl_2_, even though a 300 μM CaCl_2_ was sufficient to reach 50% of the maximal activity (Verlotta et al., 2013) (see Table 7).

The differences in the activity reported from many authors for plant sPLA_2_s is not easy to compare in absolute terms. Usually the reported activity values are informed as specific activities (μmol of hydrolyzed lipid.min^–1^.mg of protein^–1^) and this quantity may be affected by many factors. Among the main factors that can affect the sPLA_2_ activity can be mentioned (i) inherent deficiencies in the folding of recombinant enzymes, (ii) additional tags at the N-terminus, and (iii) the lack of standardization of substrate offered to the enzyme (lipid monolayers, micelles, SUVs, presence of detergents mixed with the lipid substrate, etc.). However, taking into account these precautions the activity of the reported enzymes could be compared although different substrates and systems were used in the assays (see Table 4).

### Conformational Stability of sPLA_2_s

It is known that CYS residues are essential for the structural stability of sPLA_2_ and it has been shown to play an important role in the structural stability of the mature enzyme (Six and Dennis, 2000; Welker et al., 2011). In animals, sPLA_2_s contain between 10 and 16 CYS that have the potential to form 5–8 intramolecular disulfide bridges (Schaloske and Dennis, 2006). In contrast, all sPLA_2_s reported from plants have 12 CYS that can form 6 disulfide bridges (see Table 1). It is known that some sPLA_2_ from animals (especially type I and II), are rather stable upon heating compared with cytoplasmic cPLA_2_ (Mazereeuw-Hautier et al., 2000). Resistance to heating for sPLA_2_ from plants was reported for some enzymes indicating a similar behavior to that observed for animal sPLA_2_. The structural stability for durum wheat sPLA_2_ was demonstrated by the resistance to high temperatures (87% of the activity was retained after treatment of the crude leaf extract at 100°C for 15 min), see (Verlotta et al., 2013). Recombinant *At*sPLA_2_α and *At*sPLA_2_β retained 80–95% of their activities following 5 min treatment in boiling water (Lee et al., 2005), and a similar result was obtained for sPLA2 purified from elm seeds (Stahl et al., 1998). Moreover, for *Gm*sPLA_2_-XIA-I and *Gm*sPLA_2_-XIB-II preserved the activity after heating 5 min at 80°C (Mariani et al., 2015b).

The main reason for the scarceness of information on recombinant plant sPLA_2_s may be attributed to the low expression yields obtained with the different protocols currently used and the strong propensity of the recombinant enzymes to aggregate (Mansfeld et al., 2006). The generally lower yields of the purified enzymes from inclusion bodies might be an indication for a higher fraction of misfolded and/or aggregated protein after the renaturation process. This may be the reason of different V_max_ or specific activity values obtained when studying kinetic parameters in sPLA_2_ recombinant enzymes from plants (see Table 9).

In bee venom sPLA_2_ (*Bv*sPLA_2_), it was reported that the formation of disulfide bonds is not essential for correct re-folding of the protein and an active enzyme form can be reobtained even from the completely denatured and reduced state (Welker et al., 2011). It is known that, in contrast to the seven disulfide bonds present in porcine pancreas enzyme (*Pp*sPLA_2_), all five disulfide bonds of *Bv*sPLA_2_ are essential for conformational stability and contribute to the activity (Welker et al., 2011). In the case of bacterial sPLA_2_ from *Streptomyces violaceoruber*, it possesses only two disulfide bridges (Sugiyama et al., 2002) which were sufficient to be active comparable to animal or plant sPLA_2_s (Yunes Quartino et al., 2015).

In sPLA_2_ from *A. thaliana*, the removal of disulfide bonds increased the proteolytic susceptibility of the native proteins whereas the stability decreased (Mansfeld et al., 2014). Regarding *Gm*sPLA_2_s, it was demonstrated that the calcium ion also contributes to keep the protein folded in its native structure. This effect was observed by two independent assays using dynamic simulations and intrinsic fluorescence experiments (Mariani et al., 2012, 2015b).

The comparison of the data obtained on *bovine pancreatic* sPLA_2_, *bee venom* sPLA_2_, and *porcine pancreatic* sPLA_2_ with those obtained on sPLA_2_s from plants suggests that conserved disulfide bonds in those homologous proteins are important to keep the conformational architecture and stability. However, with the recompiled information, it is almost clear that not all the disulfide bridges are needed for the protein to be active, but are necessary for a protein correct folding.

## Interfacial Catalysis Activation

Phospholipids are constituents of biological membranes, so a very important prerequisite step to perform the lipolytic action of sPLA_2_ is the interaction with the amphipathic nature of these interfaces; and in turn, determine the catalytic properties of the organized substrate (Jain and Berg, 2006). The interfacial binding step is crucial for enzymatic action of sPLA_2_, and it is mediated by a region of the protein often referred to as *i-face* (see above), also reported as *IRS, the interfacial recognition site* (Tatulian, 2001). The *i-face* or IRS is not a proper “site” or a flat face, it is rather a 3D domain with the confluence of several residues that crowns and precedes the catalytic site, giving an adequate environment for the catalysis, and also help keeping the enzyme attached to the membrane where the hydrolytic reaction takes place. The proper intimate contact of the *i-face* of sPLA_2_s with the interface is essential to provide the substrate access to the active site. Interfacial activation is a concept that means an adequate contact between the catalytic active site and the *i-face* modulating the catalytic activity (Scott et al., 1990; Tatulian et al., 2005; Jain and Berg, 2006; Winget et al., 2006). The binding and kinetic characteristics of interfacial catalysis by sPLA_2_ depend upon the organization and dynamics of the interface. The overall rate of catalytic turnover is not only determined by the kinetics at the interface, but also by the *binding/desorption equilibrium kinetics* of the enzyme with the interface (Ramirez and Jain, 1991). Hence, the hydrolysis of the organized substrate can occur in two extreme distinct modes: (i) in the *scooting mode* of catalysis, that requires that the enzyme remains bound at the interface between several catalytic turnover cycles and, (ii) in the pure *hopping mode*, where the binding and the desorption of the bound enzyme occur during each catalytic turnover cycle leading to a jumping mechanism (Jain et al., 2009) (see Supplementary Figure S4 for more details and a schematic description of both mechanism of lipids hydrolysis induced by sPLA_2_).

A few mode of interfacial catalysis for sPLA_2_s has been reported. Moreover, in plants, we were the only in studying the catalytic mode till today. The enzyme studied in order to determine the mode of catalysis was the sPLA_2_ from *G. max* (*Gm*sPLA_2_-XIA-I) (Mariani et al., 2015b). Whereas pancreatic sPLA_2_ presents a scooting mode of catalysis when using anionic lipids (Berg et al., 1991), it presents a hopping mode of catalysis if the specific experimental conditions are changed to zwitterionic lipids (Scott et al., 1994). In our hands, *Gm*sPLA_2_-XIA-I acts in the hopping mode against zwitterionic lipids (Mariani et al., 2015b).

### Hydrolysis Using Micelles as Substrate Membrane Model System

There have been some reports in the literature regarding sPLA_2_ activity against different substrates and in different conditions. For some sPLA_2_, it has been demonstrated that the hydrolysis rate is sensitive to the surface charge density of the lipid aggregates (Volwerk et al., 1986). Several kinetics studies on pancreatic as well as snake venoms and plants phospholipases have been reported in which lipid phase transition, lipid membrane curvature, and composition may modulate the lipolysis (Wilschut et al., 1978; Bell and Biltonen, 1989; Bell et al., 1996; Leidy et al., 2004). However, it should not be forgotten, that sPLA_2_ has optimum of lipid packing for hydrolysis, i.e., that some enzymes have the ability to hydrolyze lipid in a low packing organization (low lateral pressure in lipid monolayers more compatible with micelles) but others also have optimum condition of hydrolysis at high lateral pressure in monolayers compatible with liposomes or biological membranes (Ramirez and Jain, 1991; Yunes Quartino et al., 2015).

Usually, short-chain zwitterionic phospholipids have been employed as substrates in single component systems (de Haas et al., 1971; Wells, 1972) or, for the case of long-chain phospholipids, they were mixed with neutral detergents (Dennis, 1973b; Yu et al., 1999a; Mansfeld and Ulbrich-Hofmann, 2007). Moreover, the activity is generally increased when the lipid substrate forms mixed micelles in presence of detergents (Dennis, 1973a; Dennis et al., 1981). The effect of enzyme immobilization on the sPLA_2_ kinetics was also reported (Madoery et al., 1999). Description and kinetics properties of sPLA_2_ from plants have been more frequent in their recombinant counterpart after appropriate expression, purification, and folding protocols (Bahn et al., 2003; Ryu et al., 2003; Fujikawa et al., 2005; Mansfeld and Ulbrich-Hofmann, 2007; Mariani et al., 2012) compared with their equivalent found in animals sPLA_2_s. The reason of this is due to the relative high amounts of the latter proteins found in their respective natural sources (venoms and pancreatic juice) and, therefore it allows an efficient purification of the mature forms of sPLA_2_. However, few studies using purified plant enzymes were reported from elm seeds (Stahl et al., 1998) and of *G. max* (Minchiotti et al., 2008).

Mammalian and plant enzymes differed in head group specificity. While some mammalian sPLA_2_s show high activity on anionic phospholipids (Ghomashchi et al., 1991; Bezzine et al., 2002), sPLA_2_s from plants preferred zwitterionic phospholipids (Mansfeld and Ulbrich-Hofmann, 2007; Mansfeld, 2009; Mariani et al., 2015b). In Table 8 we summarize the substrate lipid preference (head group or acyl chain) differences observed in some sPLA_2_s from plants reported in the literature (see also Table 9 additional kinetic data).

**TABLE 8:** Substrate preference of different sPLA_2_s from plants.

**Origin**	**Name**	***sn*-specificity**	**Fatty acid preference**	**Head group selectivity**	**References**
*A. thaliana* (arabidopsis)	*At*sPLA_2_-α *At*sPLA_2_-β	*sn-2 sn-2*	Linoleic Palmitic> linoleic	PC>PE (baja) > < *cps*:*it* > *PG* < /*cps*:*it*≫PI PE (low)	Lee et al., 2005; Mansfeld and Ulbrich-Hofmann, 2007; Lee et al., 2005
	*At*sPLA_2_-γ	*sn-2*	Linoleic>palmitic-oleic	PE (high)	Bahn et al., 2003; Lee et al., 2005
	*At*sPLA_2_-δ	*sn-2*	Palmitic-oleic>linoleic	PE (high)	Bahn et al., 2003; Lee et al., 2005
*D. caryophillus* (carnation)	*Dc*sPLA_2_	NR	NR	NR	NR
*R. communis* (castor bean)	*Rc*sPLA2α	*sn-2*	Palmitic>ricinoleic	PC	Bayon et al., 2015
*C. sinensis* (orange)	*Cs*sPLA2α	*Deducted sn-2*	NR	NR	Liao and Burns, 2010
	*Cs*sPLA2β	*Deducted sn-2*	NR	NR	
*U. glabra* (elm)	*Ug*sPLA_2_	*sn-2*	Oleic (C8–C12)	NR	Stahl et al., 1998; Lee et al., 2005
*G. max*	*Gm*sPLA_2_-XIA-I	*sn-2*	Lauroil	PC	Mariani et al., 2015b
(soybean)	*Gm*sPLA_2_-XIA-II	*Deducted sn-2*	NR	NR	Mariani et al., 2015b
	*Gm*sPLA_2_-XIB-I	*Deducted sn-2*	NR	NR	Mariani et al., 2015b
	*Gm*sPLA_2_-XIB-II	*sn-2*	Lauroil	PC	Mariani et al., 2015b
	*Gm*sPLA_2_-XIB-III	*Deducted sn-2*	NR	NR	Mariani et al., 2015b
*L. usitatissimum*	*Lu*sPLA_2_-I	*Deducted sn-2*	NR	NR	Gupta and Dash, 2017
(flax)	*Lu*sPLA_2_-II	*Deducted sn-2*	NR	NR	Gupta and Dash, 2017
*P. somniferum* (opium)	*Ps*sPLA_2_	*sn-2*	Linolenic	PC>PE	Jablonicka et al., 2016
*O. sativa* (rice)	*Os*sPLA_2_-I	*sn-2*	NR	PC	Stahl et al., 1999;
	*Os*sPLA2-II	*sn-2*	NR	PC	Lee et al., 2005
*N. tabacum*	*Nt*1sPLA_2_	*sn-1/sn-2*	NR	PC	Fujikawa et al., 2011
(tobacco)	*Nt*2sPLA_2_	*sn-2*	NR	PC	Fujikawa et al., 2011
*L. esculentum* (tomato)	*Le*sPLA_2_	*sn-2*	NR	PC	Narvaez-Vasquez et al., 1999
*T. durum*	TdsPLA_2_-I	*sn-2*	*Non-specified*	PC	Verlotta et al., 2013
	TdsPLA_2_-II	*sn-2*	*Non-specified*	PC	
(durum wheat)	TdsPLA_2_-III	*sn-2*	Palmitic	PC	Verlotta and Trono, 2014
	TdsPLA_2_-IV	*sn-2*	*Non-specified*	PC	Verlotta et al., 2013

*NR, not reported.*

Table 9 shows the K_m_ and V_max_ values determined and reported for sPLA_2_s from different sources. As shown, we can infer that the values of V_max_ could be sensitive to both the lipid substrate used in the assays and the interfacial quality of the surface in which the substrate is inserted.

**TABLE 9:** Kinetic parameters for some reported plant sPLA_2_.

**Origin**	**K_m_ (mM)**	**V_max_ (μmol.min^–1^ mg^–1^)**	**Lipid substrate used**	**References**
*Gm*sPLA_2_-XIA-I	0.23	10.2	DLPC	Mariani et al., 2015b
	17.9	13.9	DLPG	Mariani et al., 2015b
*Gm*sPLA_2_-XIB-II	0.07	19.7	DLPC	Mariani et al., 2015b
	1.1	6.7	DLPG	Mariani et al., 2015b
*At*sPLA_2_-α	5.7	29.8	DOPC	Mansfeld and Ulbrich-Hofmann, 2007
*Td*sPLA_2_	0.43	1.43 U.g^–1a^	PC	Verlotta et al., 2013
Reported for animal sPLA_2_s	0.18–3.2	NR	DOPC	Mansfeld and Ulbrich-Hofmann, 2007
*Pp*sPLA_2_^b^	3.7	2	diC8-PC	Kuipers et al., 1991

*K_*m*_ is expressed as specific activity. V_*max*_ is expressed as μmol.min^–1^.mg^–1^. ^*a*^Expressed in Units per gram of dry leaves extract. ^*b*^Pig pancreatic sPLA_2_.
*

### Phospholipid Hydrolysis Using Langmuir Monolayers as Membrane Model System

The influence of substrate lipid packing on sPLA_2_s activities was studied for numerous authors using Langmuir-lipid monolayers performed at different surface pressures using almost exclusively sPLA_2_ from animal sources (Yunes Quartino et al., 2015). Moreover, to study the catalytic activity at the air-water interface the lipid monolayer technique in the “zero order” regime was used since the surface pressure is kept constant during the reaction (Panaiotov and Verger, 2000; Yunes Quartino et al., 2012) (see Supplementary Figure S3 for a schematic representation of this experimental system).

The optimum surface pressure of these enzymes to hydrolyze the lipids of the membranes differed with the origin of the sPLA_2_ (Ramirez and Jain, 1991; Mariani et al., 2015b; Yunes Quartino et al., 2015). *Gm*sPLA_2_s were the first sPLA_2_s from plants to be studied with respect to their interfacial characteristics. Table 10 shows the optimum pressure determined for different sPLA_2_s. The optimum for plants *Gm*sPLA_2_s seems to fall intermediate in between the values of “pancreatic like” enzymes that have high activity against micelles structured lipids rather bilayers (lipolytic ratio lower than 0.1) compared with toxic venom sPLA_2_s (lipolytic ratio higher than 1) that can hydrolyze intact cell membranes such as erythrocytes (Demel et al., 1975). Then, it may be concluded that sPLA_2_s from plants would have a more ubiquitous functionality, since they can be active *in vitro* against a rather wide range of curvature radio of structured lipid substrates (less sensitivity to the supramolecular organization).

**TABLE 10:** Parameters determined for different sPLA_2_s using monomolecular films of DLPC.

**Phospholipase A_2_ origin**	**Optimum surface pressure**	**Substrate**	**Lipolytic ratio LR_(20/10)_**	**References**
*Gm*sPLA_2_-XIA-I	13	DLPC	0.45	This review
*Gm*sPLA_2_-XIB-II	16	DLPC	0.25	This review
*B. diporus* sPLA_2_-I	11	DLPC	∼0	Yunes Quartino et al., 2015
*B. diporus* sPLA_2_-II	12	DLPC	∼0	Yunes Quartino et al., 2015
*M. fulvius*-12	9–10	DLPC	0.07	Fernandez et al., 2017
Pig pancreas *Pp*sPLA_2_	9	DLPC	0.08	Yunes Quartino et al., 2015
Bee venom *Bv*sPLA_2_	18	DLPC	1.1	Yunes Quartino et al., 2015
*B. diporus Bd*sPLA_2_-III	20	DLPC	1.3	Yunes Quartino et al., 2015
*B. asper Bs*sPLA_2_-III	18	DLPC	1.3	Yunes Quartino et al., 2015
*N. naja Nn*sPLA_2_	17	DLPC	1.5	Yunes Quartino et al., 2015
*N. m. mossambica Nm*sPLA_2_	18	DLPC	1.6	Yunes Quartino et al., 2015
*M. fulvius*-17	19–20	DLPC	1.7	Fernandez et al., 2017

### Auxin Effect Over sPLA_2_ Activity

Studies of plant sPLA_2_s demonstrated that auxins play important roles in signal transduction regulating cellular processes and probably they are implicated in phospholipid signaling (Wang, 2001; Ryu et al., 2005; Scherer et al., 2010). At the cellular level, auxins control cell division, growth, extension, and differentiation (Davies, 1995). At the whole plant level, auxins play an essential role in processes such as apical dominance, lateral/adventitious root formation, tropisms, fruit set and development, vascular differentiation, and embryogenesis (Friml, 2003). A rapid increase in sPLA_2_ activity was first verified by treating isolated microsomes and cell cultures with auxins (Scherer and Andre, 1989; Scherer, 1990; Andre and Scherer, 1991; Scherer, 1992; Scherer and Andre, 1993; Scherer, 1996) and microsomes isolated from hypocotyls segments (Blanchet et al., 2008b). However, as the molecular mechanism of the putative effect of auxins over sPLA_2_s is unknown we have investigated whether these phytohormone have any direct effect over the enzyme by using simple *in vitro* assays.

Secretory phospholipases, like other lipolytic enzymes, are interfacial active proteins, since they access from water to the interface of the insoluble organized substrate to carry out the lipid hydrolysis. For this reason, the activity of the enzyme is directly modulated at the interface by the supramolecular organization of the substrate summarized in the concept of “*interfacial quality*” [e.g., the physical state of the lipids, proper lateral packing, modulation by non-substrate lipids, “membrane lateral defects,” among others (Verger et al., 1978; Daniele et al., 1996; Jain and Berg, 2006; Blanchet et al., 2008a; Campagnoli et al., 2008; Fico et al., 2008; De Tullio et al., 2013)].

The stimulation effect of auxins over recombinant sPLA_2_s from *G. max* is rather an interfacial effect. Despite porcine pancreas sPLA_2_ presents low identity with the known reported sPLA_2_s from plant sources, shows a significant similarity in the active site and calcium binding loop regions (Mansfeld and Ulbrich-Hofmann, 2007), making it an acceptable model for comparison. Using mixed micelles was determined that the effect of auxins on sPLA_2_ stimulation depends on the concentration of the phytohormones employed with an optimal effect around 100 μM [the maximum perturbing effect (Mariani et al., 2015a)]. The hypotheses states that a direct action over sPLA_2_ enzyme molecule or a synergic effect on the micelle surface doing more favorable the interface for lipolysis occurs. Both phytohormones IAA (indole 3-acetic acid) and IPA (indole 3-propionic acid) were active toward both type of sPLA_2_, either coming from plant or pancreatic sPLA_2_, suggesting that there is not a direct specific enzyme-phytohormone interaction involved. So, the effect of auxins can be attributable to changes in the interfacial quality of the organized substrate rather than a direct effect over the enzyme (Mariani et al., 2015a). The molecular details by which the particular mixed interfaces formed by auxins/phospholipids may modulate the sPLA_2_ activity, regardless of the enzyme origin, remain to be elucidated. However, to ascertain the interfacial hypothesis of auxins over the action of sPLA_2_ we further analyzed the surface properties of two auxins: IAA and IPA, i.e., the capability of these phytohormones to partition into lipid interfaces (Mariani et al., 2015a). Both IAA and IPA did not show any affinity toward lipid-clean interfaces (self-adsorption to water surface) but, very importantly, both auxins showed *the ability to penetrate lipid interfaces* forming stable and insoluble monolayers with phospholipids. This capability to form mixed lipid-auxin interfaces allowed the activation of two recombinants *Gm*sPLA_2_s and pancreatic sPLA_2_ (Mariani et al., 2015a). The interfacial activation exerted by auxins was, regardless of sPLA_2_ source, supporting the theory that at the action is at lipid-auxin interface and not a direct effect over the enzyme (Mariani et al., 2015a).

## Potential Industrial Application and Perspectives

The application of biotechnology, particularly enzymes in industrial processes, is continuously growing due to its minimal environmental impact, since they produce non-toxic waste substances and consume little energy (Warner, 2005). Natural and modified phospholipids have been extensively used in food industry, cosmetics, pharmaceuticals and agriculture (Guo et al., 2005). Therefore, in the production of these “modified phospholipids,” secreted phospholipases obtained mostly from microorganisms or mammals have been used by the industry either for refined oils, dairy products, baked goods and other health food industries (De Maria et al., 2007; Wang et al., 2012). As sPLA_2_ enzyme catalyze the stereospecific hydrolysis at the chiral carbon (*sn-2*) of glycerophospholipids converting them to lysoderivatives, the enzymatic bioconversion is the only selective pathway for obtaining *sn-*2-lysophospholipids. Lysophospholipids have a greater bioemulsifiers capability and have been applied in food and pharmaceutical industries (Stafford and Dennis, 1988). In this regard, most sPLA_2_s used are from animal pancreas (porcine or bovine) or venoms (bee, snake) since they are enzymes easily isolated in large quantities relatively to the low cost and they are commercially available (de Haas et al., 1968; De Maria et al., 2007). However, products of animal source, are rejected by many customers for religious reasons or risk of viral or prion contamination. Moreover, the use of enzymes from animal sources in processes for obtaining food additives may be incompatible with certain international regulations, which is not accepted in certain fields of application, since they do not meet the requirements of current international food standards. This is the reason why the industrial production of vegetable sPLA_2_s may become desirable. Nevertheless microbial sPLA_2_s are being accepted, sPLA_2_s from plant would be an advantage because its putative natural specificity (Lee et al., 2005; Mansfeld, 2009).

In the last decade, research has focused on the study of the still little known vegetable sPLA_2_s (Wang, 2001). Important advances have taken place in the identification, classification, biochemical characterization and functional analysis of plant sPLAs. Recent progress in understanding the biochemical and functional properties of plant sPLAs paves the way for approval of them for commercial use and various applications. Several sPLA_2_s have shown great potential as a target in the field of plant biotechnology, and molecular and catalytic diversity of plant sPLA_2_s shows that the phospholipases are of increasing value for biotechnology applications.

The possibility of using plant phospholipases in food processing would be an advantage, from the point of view of food regulations. Considering the large production of soybean in the world, it is of great interest to study the properties of its lipolytic enzymes in terms from of both agronomic and biotechnology point of views (Rönner, 2003; Hermida, 2005). Moreover, it should be noted that in the purification process of soybean oil, a byproduct named “gum” is a material enriched in phospholipids (about 65% of dry weight), which is usually used in animal’s food production or, after drying, it is sold as *soybean lecithin*. The hydrolytic products obtained by the action of sPLA_2_ over soybean lecithin, the lysophospholipids (lysoderivatives), are widely used as emulsifiers (Henderson et al., 1995; Dashiell, 2001).

Recently, sPLA_2_s were tested as catalysts for the synthesis of phospholipids with defined fatty acids by transesterification of lysophospholipids (Mansfeld, 2009). Furthermore, plant sPLA_2_s showed to be distinctive from animals due to differences in substrate selectivity regarding the polar head and the acyl chains of glycerophospholipids (Lee et al., 2005). The potential properties of plant sPLAs would open new horizons to the engineering of biocatalysts.

The plant sPLA_2_s is expected to have advantages over from animals regarding the performance or the incorporation of polyunsaturated fatty acids such as linoleic acid in egg PC for food production. Therefore, the processes for the production of phospholipids with fatty acids are not common and special performance requirements are desirable. Often, small differences in primary or 3D structure result in differences in the catalytic properties, which can be of great importance in biocatalytic applications. However, despite their enormous potential, plant enzymes have not been yet considered for industrial application. This could be attributed to the limited availability of these enzymes, recently discovered and characterized. Besides, these enzymes are much less abundant in the natural environment and no plant enzymes are available commercially.

Over 100 years, experiments with members of the sPLA_2_ superfamily have been carried out and kinetic and structural characterization established sPLA_2_ as an important model of interfacial enzymology. The future of this promising enzymes seems to be very exciting, leading to find out specific inhibitors of them, and further elucidating plants sPLA_2_’s roles in cellular processes, along with potential uses in the industry.

## Author Contributions

MM and GF conceived the main idea, designed the general format of this manuscript, created the tables, and carried out the final corrections of this manuscript. MM prepared the figures and drafted this manuscript.

## Conflict of Interest Statement

The authors declare that the research was conducted in the absence of any commercial or financial relationships that could be construed as a potential conflict of interest.

## Footnotes

1 http://smart.embl-heidelberg.de/

2 https://www.rcsb.org/

3 http://srv00.recas.ba.infn.it/PMDB/main.php

## References

[B1] Anderson L. A. Dufton M. J. (1997). The action of *Taiwan cobra* venom on methionine enkephalin: a useful assay for oligopeptidase content. *Toxicon* 35 1113–1123. 10.1016/S0041-0101(97)00007-X 9248009

[B2] Andre B. Scherer G. F. (1991). Stimulation by auxin of phospholipase A in membrane vesicles from an auxin-sensitive tissue is mediated by an auxin receptor. *Planta* 185 209–214. 10.1007/BF00194062 24186343

[B3] Annand R. R. Kontoyianni M. Penzotti J. E. Dudler T. Lybrand T. P. Gelb M. H. (1996). Active site of bee venom phospholipase A2: the role of histidine-34, aspartate-64 and tyrosine-87. *Biochemistry* 35 4591–4601. 10.1021/bi9528412 8605210

[B4] Assmann S. M. Shimazaki K. (1999). The multisensory guard cell. Stomatal responses to blue light and abscisic acid. *Plant Physiol.* 119 809–816. 10.1104/pp.119.3.809 10069820 PMC1539207

[B5] Bahn S. C. Lee H. Y. Kim H. J. Ryu S. B. Shin J. S. (2003). Characterization of Arabidopsis secretory phospholipase A2-gamma cDNA and its enzymatic properties. *FEBS Lett.* 553 113–118. 10.1016/S0014-5793(03)00982-7 14550557

[B6] Bahnson B. J. (2005). Structure, function and interfacial allosterism in phospholipase A2: insight from the anion-assisted dimer. *Arch. Biochem. Biophys.* 433 96–106. 10.1016/j.abb.2004.08.013 15581569

[B7] Bai S. Jain M. K. Berg O. G. (2008). Contiguous binding of decylsulfate on the interface-binding surface of pancreatic phospholipase A2. *Biochemistry* 47 2899–2907. 10.1021/bi702164n 18260608

[B8] Baynes J. W. Marek D. H. (2004). *Medical Biochemistry.* Maryland Heights, MO: Mosby.

[B9] Bayon S. Chen G. Weselake R. J. Browse J. (2015). A small phospholipase A(2)-α from castor catalyzes the removal of hydroxy fatty acids from phosphatidylcholine in transgenic Arabidopsis Seeds. *Plant Physiol.* 167 1259–1270. 10.1104/pp.114.253641 25667315 PMC4378157

[B10] Bell J. D. Biltonen R. L. (1989). Thermodynamic and kinetic studies of the interaction of vesicular dipalmitoylphosphatidylcholine with *Agkistrodon piscivorus* piscivorus phospholipase A2. *J. Biol. Chem.* 264 225–230. 2909516

[B11] Bell J. D. Burnside M. Owen J. A. Royall M. L. Baker M. L. (1996). Relationships between bilayer structure and phospholipase A2 activity: interactions among temperature, diacylglycerol, lysolecithin, palmitic acid, and dipalmitoylphosphatidylcholine. *Biochemistry* 35 4945–4955. 10.1021/bi952274i 8664287

[B12] Berg O. G. Gelb M. H. Tsai M. D. Jain M. K. (2001). Interfacial enzymology: the secreted phospholipase A(2)-paradigm. *Chem. Rev.* 101 2613–2654. 10.1021/cr990139w 11749391

[B13] Berg O. G. Yu B. Z. Jain M. K. (2009). Thermodynamic reciprocity of the inhibitor binding to the active site and the interface binding region of IB phospholipase A2. *Biochemistry* 48 3209–3218. 10.1021/bi801244u 19301847

[B14] Berg O. G. Yu B. Z. Rogers J. Jain M. K. (1991). Interfacial catalysis by phospholipase A2: determination of the interfacial kinetic rate constants. *Biochemistry* 30 7283–7297. 10.1021/bi00243a034 1854737

[B15] Bezzine S. Bollinger J. G. Singer A. G. Veatch S. L. Keller S. L. Gelb M. H. (2002). On the binding preference of human groups IIA and X phospholipases A2 for membranes with anionic phospholipids. *J. Biol. Chem.* 277 48523–48534. 10.1074/jbc.M203137200 12244093

[B16] Blanchet M. H. Le Good J. A. Mesnard D. Oorschot V. Baflast S. Minchiotti G. (2008a). Cripto recruits Furin and PACE4 and controls nodal trafficking during proteolytic maturation. *EMBO J.* 27 2580–2591. 10.1038/emboj.2008.174 18772886 PMC2567404

[B17] Blanchet M. H. Le Good J. A. Oorschot V. Baflast S. Minchiotti G. Klumperman J. (2008b). Cripto localizes nodal at the limiting membrane of early endosomes. *Sci. Signal.* 1:ra13. 10.1126/scisignal.1165027 19001664

[B18] Burke J. E. Dennis E. A. (2009). Phospholipase A2 structure/function, mechanism, and signaling. *J. Lipid Res.* 50(Suppl.), S237–S242. 10.1194/jlr.R800033-JLR200 19011112 PMC2674709

[B19] Campagnoli M. Hansson P. Dolcini L. Caridi G. Dagnino M. Candiano G. (2008). Analbuminemia in a swedish male is caused by the kayseri mutation (c228_229delAT). *Clin. Chim. Acta* 396 89–92. 10.1016/j.cca.2008.06.008 18602380

[B20] Catanzariti A. M. Soboleva T. A. Jans D. A. Board P. G. Baker R. T. (2004). An efficient system for high-level expression and easy purification of authentic recombinant proteins. *Protein Sci.* 13 1331–1339. 10.1110/ps.04618904 15096636 PMC2286746

[B21] Chen G. Snyder C. L. Greer M. S. Weselake R. J. (2011). Biology and biochemistry of plant phospholipases. *Crit. Rev. Plant Sci.* 30 239–258. 10.1080/07352689.2011.572033

[B22] Chiou Y. L. Cheng Y. C. Kao P. H. Wang J. J. Chang L. S. (2008). Mutations on the N-terminal region abolish differentially the enzymatic activity, membrane-damaging activity and cytotoxicity of Taiwan cobra phospholipase A2. *Toxicon* 51 270–279. 10.1016/j.toxicon.2007.10.001 18022206

[B23] Choi M. S. Rhee K. C. (2006). Production and processing of soybeans and nutrition and safety of isoflavone and other soy products for human health. *J. Med. Food* 9 1–10. 10.1089/jmf.2006.9.1 16579721

[B24] Daniele J. J. Bianco I. D. Delgado C. Carrillo D. B. Fidelio G. D. (1997). A new phospholipase A2 isoform isolated from *Bothrops neuwiedii* (*Yarara chica*) venom with novel kinetic and chromatographic properties. *Toxicon* 35 1205–1215. 10.1016/S0041-0101(97)00023-8 9278970

[B25] Daniele J. J. Maggio B. Bianco I. D. Goni F. M. Alonso A. Fidelio G. D. (1996). Inhibition by gangliosides of *Bacillus cereus* phospholipase C activity against monolayers, micelles and bilayer vesicles. *Eur. J. Biochem.* 239 105–110. 10.1111/j.1432-1033.1996.0105u.x 8706693

[B26] Dashiell G. (2001). Fuentes, métodos de procesos y usos comerciales de lecitina. *Aceites Grasas* 43 197–204.

[B27] Davies P. J. (1995). “The plant hormones: their nature, occurrence, and functions,” in *Plant Hormones: Physiology, Biochemistry and Molecular Biology*, ed. Davies P. J. (Boston, MA: Kluwer), 13–38.

[B28] de Haas G. H. Bonsen P. P. Pieterson W. A. van Deenen L. L. (1971). Studies on phospholipase A and its zymogen from porcine pancreas. 3. Action of the enzyme on short-chain lecithins. *Biochim. Biophys. Acta* 239 252–266. 10.1016/0005-2760(71)901718 5165845

[B29] de Haas G. H. Postema N. M. Nieuwenhuizen W. van Deenen L. L. (1968). Purification and properties of phospholipase A from porcine pancreas. *Biochim. Biophys. Acta* 159 103–117. 10.1016/0005-2744(68)90248-9 4967810

[B30] De Maria L. Vind J. Oxenboll K. M. Svendsen A. Patkar S. (2007). Phospholipases and their industrial applications. *Appl. Microbiol. Biotechnol.* 74 290–300. 10.1007/s00253-006-0775-x 17221199

[B31] De Tullio L. Fanani M. L. Maggio B. (2013). Surface mixing of products and substrate of PLA(2) in enzyme-free mixed monolayers reproduces enzyme-driven structural topography. *Biochim. Biophys. Acta* 1828 2056–2063. 10.1016/j.bbamem.2013.05.018 23727527

[B32] Demel R. A. Geurts van Kessel W. S. Zwaal R. F. Roelofsen B. van Deenen L. L. (1975). Relation between various phospholipase actions on human red cell membranes and the interfacial phospholipid pressure in monolayers. *Biochim. Biophys. Acta* 406 97–107. 10.1016/0005-2736(75)900450 1174576

[B33] Dennis E. A. (1973a). Kinetic dependence of phospholipase A 2 activity on the detergent Triton X-100. *J. Lipid Res.* 14 152–159. 4698263

[B34] Dennis E. A. (1973b). Phospholipase A2 activity towards phosphatidylcholine in mixed micelles: surface dilution kinetics and the effect of thermotropic phase transitions. *Arch. Biochem. Biophys.* 158 485–493. 10.1016/0003-9861(73)905407 4798723

[B35] Dennis E. A. Cao J. Hsu Y. H. Magrioti V. Kokotos G. (2011). Phospholipase A2 enzymes: physical structure, biological function, disease implication, chemical inhibition, and therapeutic intervention. *Chem. Rev.* 111 6130–6185. 10.1021/cr200085w 21910409 PMC3196595

[B36] Dennis E. A. Darke P. L. Deems R. A. Kensil C. R. Pluckthun A. (1981). Cobra venom phospholipase A2: a review of its action toward lipid/water interfaces. *Mol. Cell. Biochem.* 36 37–45. 10.1007/BF02354830 7242529

[B37] Dhondt S. Geoffroy P. Stelmach B. A. Legrand M. Heitz T. (2000). Soluble phospholipase A2 activity is induced before oxylipin accumulation in tobacco mosaic virus-infected tobacco leaves and is contributed by patatin-like enzymes. *Plant J.* 23 431–440. 10.1046/j.1365-313x.2000.00802.x 10972869

[B38] Dijkstra B. W. Kalk K. H. Drenth J. de Haas G. H. Egmond M. R. Slotboom A. J. (1984). Role of the N-terminus in the interaction of pancreatic phospholipase A2 with aggregated substrates. Properties and crystal structure of transaminated phospholipase A2. *Biochemistry* 23 2759–2766. 6466614 10.1021/bi00307a035

[B39] Dijkstra B. W. Kalk K. H. Hol W. G. Drenth J. (1981). Structure of bovine pancreatic phospholipase A2 at 1.7A resolution. *J. Mol. Biol.* 147 97–123. 10.1016/0022-2836(81)900814 7265241

[B40] Domingues S. J. S. de SouzaI T. F. Soares A. M. S. Jacinto T. Machado O. L. T. (2007). Activation of phospholipase PLA2 actvity in *Ricinus communis* leaves in response to mechanical wounding. *Braz. J. Plant Physiol.* 19 35–42.

[B41] dos Santos J. I. Cintra-Francischinelli M. Borges R. J. Fernandes C. A. Pizzo P. Cintra A. C. (2011). Structural, functional, and bioinformatics studies reveal a new snake venom homologue phospholipase A class. *Proteins* 79 61–78. 10.1002/prot.22858 20878713

[B42] Fernandez M. L. Quartino P. Y. Arce-Bejarano R. Fernandez J. Camacho L. F. Gutierrez J. M. (2017). Intravascular hemolysis induced by phospholipases A2 from the venom of the eastern coral snake, *Micrurus fulvius*: functional profiles of hemolytic and non-hemolytic isoforms. *Toxicol. Lett.* 286 39–47. 10.1016/j.toxlet.2017.11.037 29197624

[B43] Fico A. Manganelli G. Simeone M. Guido S. Minchiotti G. Filosa S. (2008). High-throughput screening-compatible single-step protocol to differentiate embryonic stem cells in neurons. *Stem Cells Dev.* 17 573–584. 10.1089/scd.2007.0130 18576914

[B44] Friml J. (2003). Auxin transport - shaping the plant. *Curr. Opin. Plant Biol.* 6 7–12. 10.1016/S1369526602000031 12495745

[B45] Fujikawa R. Fujikawa Y. Iijima N. Esaka M. (2005). Molecular cloning, expression, and characterization of secretory phospholipase A2 in tobacco. *Lipids* 40 901–908. 10.1007/s11745-005-14509 16329463

[B46] Fujikawa Y. Fujikawa R. Iijima N. Esaka M. (2011). Characterization of secretory phospholipase A(2) with phospholipase A(1) activity in tobacco, *Nicotiana tabacum* (L.). *Lipids* 47 303–312. 10.1007/s11745-011-36323 22124805

[B47] Ghomashchi F. Yu B. Z. Berg O. Jain M. K. Gelb M. H. (1991). Interfacial catalysis by phospholipase A2: substrate specificity in vesicles. *Biochemistry* 30 7318–7329. 10.1021/bi00243a037 1854740

[B48] Guo Z. Vikbjerg A. F. Xu X. (2005). Enzymatic modification of phospholipids for functional applications and human nutrition. *Biotechnol. Adv.* 23 203–259. 10.1016/j.biotechadv.2005.02.001 15763405

[B49] Gupta P. Dash P. K. (2017). Molecular details of secretory phospholipase A2 from flax (*Linum usitatissimum* L.) provide insight into its structure and function. *Sci. Rep.* 7:11080. 10.1038/s41598-017-109699 28894144 PMC5593939

[B50] Gupta P. Saini R. Dash P. K. (2017). Origin and evolution of group XI secretory phospholipase A2 from flax (*Linum usitatissimum*) based on phylogenetic analysis of conserved domains. *3 Biotech.* 7:216. 10.1007/s13205-017-0790-x 28669075 PMC5494027

[B51] Guy J. E. Stahl U. Lindqvist Y. (2009). Crystal structure of a class XIB phospholipase A2 (PLA2): rice (oryza sativa) isoform-2 pla2 and an octanoate complex. *J. Biol. Chem.* 284 19371–19379. 10.1074/jbc.M109.008466 19457861 PMC2740562

[B52] Havinga T. (2010). Regulating halal and kosher foods: different arrangements between state, industry and religious actors. *Erasmus Law Rev.* 3 241–255. 10.553/ELR221026712010003004004

[B53] Henderson J. Atkinson A. E. Lazarus C. M. Hawes C. R. Napier R. M. Macdonald H. (1995). Stable expression of maize auxin-binding protein in insect cell lines. *FEBS Lett.* 371 293–296. 10.1016/0014-5793(95)00922-V 7556613

[B54] Hermida R. (2005). Presente y futuro de la industria aceitera a nivel nacional e internacional. *Aceites Grasas* 58 38–44.

[B55] Hirel P. H. Schmitter M. J. Dessen P. Fayat G. Blanquet S. (1989). Extent of N-terminal methionine excision from *Escherichia coli* proteins is governed by the side-chain length of the penultimate amino acid. *Proc. Natl. Acad. Sci. U.S.A.* 86 8247–8251. 10.1073/pnas.86.21.8247 2682640 PMC298257

[B56] Jabeen T. Singh N. Singh R. K. Ethayathulla A. S. Sharma S. Srinivasan A. (2005). Crystal structure of a novel phospholipase A2 from Naja naja sagittifera with a strong anticoagulant activity. *Toxicon* 46 865–875. 10.1016/j.toxicon.2005.08.008 16269164

[B57] Jablonicka V. Mansfeld J. Heilmann I. Oblozinsky M. Heilmann M. (2016). Identification of a secretory phospholipase A2 from *Papaver somniferum* L. that transforms membrane phospholipids. *Phytochemistry* 129 4–13. 10.1016/j.phytochem.2016.07.010 27473012

[B58] Jain E. Bairoch A. Duvaud S. Phan I. Redaschi N. Suzek B. E. (2009). Infrastructure for the life sciences: design and implementation of the UniProt website. *BMC Bioinformatics* 10:136. 10.1186/1471-2105-10-136 19426475 PMC2686714

[B59] Jain M. K. Berg O. G. (2006). Coupling of the i-face and the active site of phospholipase A2 for interfacial activation. *Curr. Opin. Chem. Biol.* 10 473–479. 10.1016/j.cbpa.2006.08.015 16938485

[B60] Jorgensen W. L. Chandrasekhar J. Madura J. D. Impey R. W. Klein M. L. (1983). Comparison of single potential functions for simulating liquid water. *J. Chem. Phys.* 79 2577–2637. 10.1063/1.445869

[B61] Khan W. A. Blobe G. C. Hannun Y. A. (1995). Arachidonic acid and free fatty acids as second messengers and the role of protein kinase C. *Cell Signal.* 7 171–184. 10.1016/0898-6568(94)00089-T 7662506

[B62] Kim J. Y. Chung Y. S. Ok S. H. Lee S. G. Chung W. I. Kim I. Y. (1999). Characterization of the full-length sequences of phospholipase A2 induced during flower development. *Biochim. Biophys. Acta* 1489 389–392. 10.1016/S0167-4781(99)001931 10673040

[B63] Kohler G. A. Brenot A. Haas-Stapleton E. Agabian N. Deva R. Nigam S. (2006). Phospholipase A2 and phospholipase B activities in fungi. *Biochim. Biophys. Acta* 1761 1391–1399. 10.1016/j.bbalip.2006.09.011 17081801 PMC2077850

[B64] Kramer R. M. Hession C. Johansen B. Hayes G. McGray P. Chow E. P. (1989). Structure and properties of a human non-pancreatic phospholipase A2. *J. Biol. Chem.* 264 5768–5775. 2925633

[B65] Kuipers O. P. Vincent M. Brochon J. C. Verheij H. M. de Haas G. H. Gallay J. (1991). Insight into the conformational dynamics of specific regions of porcine pancreatic phospholipase A2 from a time-resolved fluorescence study of a genetically inserted single tryptophan residue. *Biochemistry* 30 8771–8785. 10.1021/bi00100a008 1888737

[B66] Kumar A. Sekharudu C. Ramakrishnan B. Dupureur C. M. Zhu H. Tsai M. D. (1994). Structure and function of the catalytic site mutant Asp 99 Asn of phospholipase A2: absence of the conserved structural water. *Protein Sci.* 3 2082–2088. 10.1002/pro.5560031121 7703854 PMC2142646

[B67] Laigle J. L. Leger H. Couraud L. Guillard J. M. Fontan D. Verger P. (1973). Pulmonary fibroxanthoma in the child. Apropos of a new case. *Pédiatrie* 28 755–763. 4803538

[B68] Lee H. Y. Bahn S. C. Kang Y. M. Lee K. H. Kim H. J. Noh E. K. (2003). Secretory low molecular weight phospholipase A2 plays important roles in cell elongation and shoot gravitropism in Arabidopsis. *Plant Cell* 15 1990–2002. 10.1105/tpc.014423 12953106 PMC181326

[B69] Lee H. Y. Bahn S. C. Shin J. S. Hwang I. Back K. Doelling J. H. (2005). Multiple forms of secretory phospholipase A2 in plants. *Prog. Lipid Res.* 44 52–67. 10.1016/j.plipres.2004.10.002 15748654

[B70] Leidy C. Mouritsen O. G. Jorgensen K. Peters G. H. (2004). Evolution of a rippled membrane during phospholipase A2 hydrolysis studied by time-resolved AFM. *Biophys. J.* 87 408–418. 10.1529/biophysj.103.036103 15240475 PMC1304362

[B71] Liao H. L. Burns J. K. (2010). Light controls phospholipase A2alpha and beta gene expression in *Citrus sinensis*. *J. Exp. Bot.* 61 2469–2478. 10.1093/jxb/erq083 20388744 PMC2877900

[B72] Liscovitch M. Czarny M. Fiucci G. Tang X. (2000). Phospholipase D: molecular and cell biology of a novel gene family. *Biochem. J.* 345(Pt 3), 401–415. 10.1042/bj3450401 10642495 PMC1220771

[B73] Lomize M. A. Pogozheva I. D. Joo H. Mosberg H. I. Lomize A. L. (2012). OPM database and PPM web server: resources for positioning of proteins in membranes. *Nucleic Acids Res.* 40 D370–D376. 10.1093/nar/gkr703 21890895 PMC3245162

[B74] Madoery R. R. Gattone C. G. Fidelio G. D. (1999). The effect of phospholipase A(2) immobilization upon calcium interaction: a kinetic study. *J. Biochem.* 126 1060–1066. 10.1093/oxfordjournals.jbchem.a022550 10578057

[B75] Mansfeld J. (2009). Plant phospholipases A2: perspectives on biotechnological applications. *Biotechnol. Lett.* 31 1373–1380. 10.1007/s10529-009-00341 19479320

[B76] Mansfeld J. Gebauer S. Dathe K. Ulbrich-Hofmann R. (2006). Secretory phospholipase A2 from *Arabidopsis thaliana*: insights into the three-dimensional structure and the amino acids involved in catalysis. *Biochemistry* 45 5687–5694. 10.1021/bi052563z 16669612

[B77] Mansfeld J. Schopfel M. Lorenz J. Trutschel T. Heilmann I. Brandt W. (2014). Probing selected structural regions in the secreted phospholipase A(2) from *Arabidopsis thaliana* for their impact on stability and activity. *Biochimie* 101 60–66. 10.1016/j.biochi.2013.12.015 24389456

[B78] Mansfeld J. Ulbrich-Hofmann R. (2007). Secretory phospholipase A2-alpha from *Arabidopsis thaliana*: functional parameters and substrate preference. *Chem. Phys. Lipids* 150 156–166. 10.1016/j.chemphyslip.2007.07.001 17692835

[B79] Mariani M. E. Madoery R. R. Fidelio G. D. (2015a). Auxins action on *Glycine max* secretory phospholipase A2 is mediated by the interfacial properties imposed by the phytohormones. *Chem. Phys. Lipids* 189 1–6. 10.1016/j.chemphyslip.2015.05.003 25987194

[B80] Mariani M. E. Madoery R. R. Fidelio G. D. (2015b). Kinetic characterization, optimum conditions for catalysis and substrate preference of secretory phospholipase A2 from *Glycine max* in model membrane systems. *Biochimie* 108 48–58. 10.1016/j.biochi.2014.10.016 25447147

[B81] Mariani M. E. Villarreal M. A. Cheung F. Leiva E. P. Madoery R. R. Fidelio G. D. (2012). In silico and in vitro characterization of phospholipase A(2) isoforms from soybean (*Glycine max*). *Biochimie* 94 2608–2619. 10.1016/j.biochi.2012.07.021 23281487

[B82] Marki F. Hanulak V. (1993). Recombinant human synovial fluid phospholipase A2 and N-terminal variant: kinetic parameters and response to inhibitors. *J. Biochem.* 113 734–737. 10.1093/oxfordjournals.jbchem.a124112 8370672

[B83] Matsushima R. Kondo M. Nishimura M. Hara-Nishimura I. (2003). A novel ER-derived compartment, the ER body, selectively accumulates a beta-glucosidase with an ER-retention signal in Arabidopsis. *Plant J.* 33 493–502. 10.1046/j.1365-313X.2003.01636.x 12581307

[B84] Mazereeuw-Hautier J. Redoules D. Tarroux R. Charveron M. Salles J. P. Simon M. F. (2000). Identification of pancreatic type I secreted phospholipase A2 in human epidermis and its determination by tape stripping. *Br. J. Dermatol.* 142 424–431. 10.1046/j.1365-2133.2000.03351.x 10735945

[B85] McIntosh J. M. Ghomashchi F. Gelb M. H. Dooley D. J. Stoehr S. J. Giordani A. B. (1995). Conodipine-M, a novel phospholipase A2 isolated from the venom of the marine snail Conus magus. *J. Biol. Chem.* 270 3518–3526. 10.1046/j.1365-2133.2000.03351.x 7876086

[B86] Meneghetti F. Maggio B. (2013). (E)-2-{[1-(3,11-Dimethyl-4-methyl-ene-10-oxo-1-phenyl-4,5,10,11-tetra-hydro-1H-be nzo[b]pyrazolo-[3,4-f][1,5]diazo-cin-5-yl)ethyl-idene]amino}-N-methyl-N-(3-methyl -1-phenyl-1H-pyrazol-5-yl)benzamide. *Acta Crystallogr. Sect. E Struct. Rep. Online* 69(Pt 10), o1583. 10.1107/S1600536813025671 24098259 PMC3790440

[B87] Messina M. J. (1999). Legumes and soybeans: overview of their nutritional profiles and health effects. *Am. J. Clin. Nutr.* 70(3 Suppl.), 439S–450S. 10.1093/ajcn/70.3.439s 10479216

[B88] Minchiotti M. Scalambro M. B. Vargas L. Coronel C. Madoery R. (2008). Isolation of phospholipase A2 from soybean (*Glycine max*) seeds: the study of its enzymatic properties. *Enzyme Microb. Technol.* 42 389–394. 10.1016/j.enzmictec.2007.11.015

[B89] Moreau R. A. Morgan C. P. (1988). Photeolytic activation of a lipolytic enzyme activity in potato leaves. *Plant Sci.* 55 205–211. 10.1016/j.enzmictec.2007.11.015

[B90] Mounier C. M. Ghomashchi F. Lindsay M. R. James S. Singer A. G. Parton R. G. (2004). Arachidonic acid release from mammalian cells transfected with human groups IIA and X secreted phospholipase A(2) occurs predominantly during the secretory process and with the involvement of cytosolic phospholipase A(2)-alpha. *J. Biol. Chem.* 279 25024–25038. 10.1074/jbc.M313019200 15007070

[B91] Mukherjee A. B. (1990). *Biochemistry, Molecular Biology and Physiology of Phospholipase A2 and its Regulatory Factors.* New York, NY: Plenum Press. 2096694

[B92] Murakami M. Sato H. Miki Y. Yamamoto K. Taketomi Y. (2015). A new era of secreted phospholipase A(2). *J. Lipid Res.* 56 1248–1261. 10.1194/jlr.R058123 25805806 PMC4479330

[B93] Murakami M. Taketomi Y. Miki Y. Sato H. Hirabayashi T. Yamamoto K. (2010). Recent progress in phospholipase A research: from cells to animals to humans. *Prog. Lipid Res.* 50 152–192. 10.1016/j.plipres.2010.12.001 21185866

[B94] Murakami M. Taketomi Y. Sato H. Yamamoto K. (2011). Secreted phospholipase A2 revisited. *J. Biochem.* 150 233–255. 10.1093/jb/mvr088 21746768

[B95] Narvaez-Vasquez J. Florin-Christensen J. Ryan C. A. (1999). Positional specificity of a phospholipase A activity induced by wounding, systemin, and oligosaccharide elicitors in tomato leaves. *Plant Cell* 11 2249–2260. 10.1105/tpc.11.11.2249 10559447 PMC144127

[B96] Othman R. Baker S. Li Y. Worrall A. F. Wilton D. C. (1996). Human non-pancreatic (group II) secreted phospholipase A2 expressed from a synthetic gene in *Escherichia coli*: characterisation of N-terminal mutants. *Biochim. Biophys. Acta* 1303 92–102. 10.1016/0005-2760(96)000835 8856038

[B97] Pagny S. Cabanes-Macheteau M. Gillikin J. W. Leborgne-Castel N. Lerouge P. Boston R. S. (2000). Protein recycling from the Golgi apparatus to the endoplasmic reticulum in plants and its minor contribution to calreticulin retention. *Plant Cell* 12 739–756. 10.1105/tpc.12.5.739 10810147 PMC139924

[B98] Pan Y. H. Yu B. Z. Berg O. G. Jain M. K. Bahnson B. J. (2002). Crystal structure of phospholipase A2 complex with the hydrolysis products of platelet activating factor: equilibrium binding of fatty acid and lysophospholipid-ether at the active site may be mutually exclusive. *Biochemistry* 41 14790–14800. 10.1021/bi026922r 12475227

[B99] Panaiotov I. Verger R. (2000). “Enzymatic reactions at interfaces: interfacial and temporal organization of enzymatic lipolysis,” in *Physical Chemistry and Biological Interfaces*, eds Baszkin A. Norde W. (Boca Raton, FL: CRC Press), 359–400.

[B100] Qin S. Pande A. H. Nemec K. N. He X. Tatulian S. A. (2005). Evidence for the regulatory role of the N-terminal helix of secretory phospholipase A(2) from studies on native and chimeric proteins. *J. Biol. Chem.* 280 36773–36783. 10.1074/jbc.M506789200 16103116

[B101] Ramirez F. Jain M. K. (1991). Phospholipase A2 at the bilayer interface. *Proteins* 9 229–239. 10.1002/prot.340090402 1866429

[B102] Randolph A. Heinrikson R. L. (1982). Crotalus atrox phospholipase A2. Amino acid sequence and studies on the function of the NH2-terminal region. *J. Biol. Chem.* 257 2155–2161. 7061414

[B103] Rhee S. G. Bae Y. S. (1997). Regulation of phosphoinositide-specific phospholipase C isozymes. *J. Biol. Chem.* 272 15045–15048. 9182519 10.1074/jbc.272.24.15045

[B104] Rönner L. (2003). *La Incorporación de Nuevas Tecnologías: el Caso de la Soja.* Buenos Aires: Universidad de Buenos Aires.

[B105] Ryu S. B. (2004). Phospholipid-derived signaling mediated by phospholipase A in plants. *Trends Plant Sci.* 9 229–235. 10.1016/j.tplants.2004.03.004 15130548

[B106] Ryu S. B. Lee H. Y. Doelling J. H. Palta J. P. (2005). Characterization of a cDNA encoding Arabidopsis secretory phospholipase A2-alpha, an enzyme that generates bioactive lysophospholipids and free fatty acids. *Biochim. Biophys. Acta* 1736 144–151. 10.1016/j.bbalip.2005.08.005 16140037

[B107] Ryu Y. Oh Y. Yoon J. Cho W. Baek K. (2003). Molecular characterization of a gene encoding the Drosophila melanogaster phospholipase A2. *Biochim. Biophys. Acta* 1628 206–210. 10.1016/S0167-4781(03)00143-X 12932833

[B108] Schaller F. (2001). Enzymes of the biosynthesis of octadecanoid-derived signalling molecules. *J. Exp. Bot.* 52 11–23. 10.1093/jxb/52.354.11 11181709

[B109] Schaloske R. H. Dennis E. A. (2006). The phospholipase A2 superfamily and its group numbering system. *Biochim. Biophys. Acta* 1761 1246–1259. 10.1016/j.bbalip.2006.07.011 16973413

[B110] Scherer G. F. (1990). Phospholipase A2 and phospholipid-activated protein kinase in plant signal transduction. *Symp. Soc. Exp. Biol.* 44 257–270. 2130515

[B111] Scherer G. F. Andre B. (1989). A rapid response to a plant hormone: auxin stimulates phospholipase A2 in vivo and in vitro. *Biochem. Biophys. Res. Commun.* 163 111–117. 10.1016/0006-291x(89)92106-2 2775252

[B112] Scherer G. F. Andre B. (1993). Stimulation of phospholipase A 2 by auxin in microsomes from suspension-cultured soybean cells is receptormediated and influenced by nucleotides. *Planta* 191 515–523.

[B113] Scherer G. F. Ryu S. B. Wang X. Matos A. R. Heitz T. (2010). Patatin-related phospholipase A: nomenclature, subfamilies and functions in plants. *Trends Plant Sci.* 15 693–700. 10.1016/j.tplants.2010.09.005 20961799

[B114] Scherer G. F. E. (1992). Stimulation of growth and phospholipase A 2 by the peptides mastoparan and melittin and by the auxin 2,4-dichlorophenoxyacetic acid. *Plant Growth Regul.* 11 153–157. 10.1007/BF00024069

[B115] Scherer G. F. E. (1996). Phospholipid signalling and lipid-derived second messengers in plants. *Plant Growth Regul.* 18 125–133. 10.1007/BF00028497

[B116] Scott D. L. Mandel A. M. Sigler P. B. Honig B. (1994). The electrostatic basis for the interfacial binding of secretory phospholipases A2. *Biophys. J.* 67 493–504. 10.1016/S0006-3495(94)80546-6 7948668 PMC1225392

[B117] Scott D. L. White S. P. Otwinowski Z. Yuan W. Gelb M. H. Sigler P. B. (1990). Interfacial catalysis: the mechanism of phospholipase A2. *Science* 250 1541–1546. 10.1126/science.2274785 2274785 PMC3443688

[B118] Seo J. Lee H. Y. Choi H. Choi Y. Lee Y. Kim Y. W. (2008). Phospholipase A2beta mediates light-induced stomatal opening in Arabidopsis. *J. Exp. Bot.* 59 3587–3594. 10.1093/jxb/ern208 18725378 PMC2561155

[B119] Shridas P. Webb N. R. (2014). Diverse functions of secretory phospholipases A2. *Adv. Vasc. Med.* 2014:11 10.1155/2014/689815

[B120] Six D. A. Dennis E. A. (2000). The expanding superfamily of phospholipase A(2) enzymes: classification and characterization. *Biochim. Biophys. Acta* 1488 1–19. 10.1016/s1388-1981(00)00105-0 11080672

[B121] Slotboom A. J. van Dam-Mieras M. C. de Haas G. H. (1977). Regulation of phospholipase A2 activity by different lipid-water interfaces. *J. Biol. Chem.* 252 2948–2951. 853038

[B122] Stafford R. E. Dennis E. A. (1988). Lysophospholipids as Biosurfactants. *Colloids Surf.* 30 47–64. 10.1016/0166-6622(87)802032 19837413

[B123] Stahl U. Ek B. Stymne S. (1998). Purification and characterization of a low-molecular-weight phospholipase A2 from developing seeds of elm. *Plant Physiol.* 117 197–205. 10.2307/4278269 9576789 PMC35004

[B124] Stahl U. Lee M. Sjodahl S. Archer D. Cellini F. Ek B. (1999). Plant low-molecular-weight phospholipase A2S (PLA2s) are structurally related to the animal secretory PLA2s and are present as a family of isoforms in rice (*Oryza sativa*). *Plant Mol. Biol.* 41 481–490. 10608658 10.1023/a:1006323405788

[B125] Sugiyama M. Ohtani K. Izuhara M. Koike T. Suzuki K. Imamura S. (2002). A novel prokaryotic phospholipase A2. Characterization, gene cloning, and solution structure. *J. Biol. Chem.* 277 20051–20058. 10.1074/jbc.M200264200 11897786

[B126] Tatulian S. A. (2001). Toward understanding interfacial activation of secretory phospholipase A2 (PLA2): membrane surface properties and membrane-induced structural changes in the enzyme contribute synergistically to PLA2 activation. *Biophys. J.* 80 789–800. 10.1016/S0006-3495(01)760584 11159446 PMC1301277

[B127] Tatulian S. A. Qin S. Pande A. H. He X. (2005). Positioning membrane proteins by novel protein engineering and biophysical approaches. *J. Mol. Biol.* 351 939–947. 10.1016/j.jmb.2005.06.080 16055150

[B128] Valentin E. Ghomashchi F. Gelb M. H. Lazdunski M. Lambeau G. (1999). On the diversity of secreted phospholipases A(2). Cloning, tissue distribution, and functional expression of two novel mouse group II enzymes. *J. Biol. Chem.* 274 31195–31202. 10.1074/jbc.274.44.31195 10531313

[B129] van Deenen L. L. (1971). Chemistry of phospholipids in relation to biological membranes. *Pure Appl. Chem.* 25 25–56. 10.1351/pac197125010025 4937801

[B130] van den Berg B. Tessari M. Boelens R. Dijkman R. de Haas G. H. Kaptein R. (1995). NMR structures of phospholipase A2 reveal conformational changes during interfacial activation. *Nat. Struct. Biol.* 2 402–406. 10.1038/nsb0595-402 7664098

[B131] van Scharrenburg G. J. Jansen E. H. Egmond M. R. de Haas G. H. Slotboom A. J. (1984). Structural importance of the amino-terminal residue of pancreatic phospholipase A2. *Biochemistry* 23 6285–6294. 10.1021/bi00320a059 6441599

[B132] Verger R. Rietsch J. Pattus F. Ferrato F. Pieroni G. De Haas G. H. (1978). Studies of lipase and phospholipase A2 acting on lipid monolayers. *Adv. Exp. Med. Biol.* 101 79–94. 10.1002/bbpc.19780820923 665391

[B133] Verlotta A. Liberatore M. T. Cattivelli L. Trono D. (2013). Secretory Phospholipases A2 in durum wheat (*Triticum durum* Desf.): gene expression, enzymatic activity, and relation to drought stress adaptation. *Int. J. Mol. Sci.* 14 5146–5169. 10.3390/ijms14035146 23455473 PMC3634499

[B134] Verlotta A. Trono D. (2014). Expression, purification and refolding of active durum wheat (*Triticum durum* Desf.) secretory phospholipase A2 from inclusion bodies of *Escherichia coli*. *Protein Expr. Purif.* 101 28–36. 10.1016/j.pep.2014.05.009 24925645

[B135] Volwerk J. J. Jost P. C. de Haas G. H. Griffith O. H. (1986). Activation of porcine pancreatic phospholipase A2 by the presence of negative charges at the lipid-water interface. *Biochemistry* 25 1726–1733. 10.1021/bi00355a042 3707906

[B136] Wang G. Ryu S. Wang X. (2012). “Plant phospholipases: an overview,” in *Lipases and Phospholipases: Methods and Protocols*, ed. Sandoval G. (New York, NY: Humana Press), 123–137. 10.1007/978-1-61779-600-5_8 22426716

[B137] Wang X. (2001). Plant phospholipases. *Annu. Rev. Plant Physiol. Plant Mol. Biol.* 52 211–231. 10.1146/annurev.arplant.52.1.211 11337397

[B138] Warner S. (2005). *Biotech takes on New Industries.* Available at: https://www.the-scientist.com/biobusiness/biotech-takes-on-new-industries-49036

[B139] Welker S. Markert Y. Koditz J. Mansfeld J. Ulbrich-Hofmann R. (2011). Disulfide bonds of phospholipase A2 from bee venom yield discrete contributions to its conformational stability. *Biochimie* 93 195–201. 10.1016/j.biochi.2010.09.012 20884319

[B140] Wells M. A. (1972). A kinetic study of the phospholipase A 2 (*Crotalus adamanteus*) catalyzed hydrolysis of 1,2-dibutyryl-sn-glycero-3-phosphorylcholine. *Biochemistry* 11 1030–1041. 10.1021/bi00756a013 5062536

[B141] West N. Newcombe R. G. Hughes N. Mason S. Maggio B. Sufi F. (2013). A 3-day randomised clinical study investigating the efficacy of two toothpastes, designed to occlude dentine tubules, for the treatment of dentine hypersensitivity. *J. Dent.* 41 187–194. 10.1016/j.jdent.2012.11.007 23160037

[B142] Williams R. L. (1999). Mammalian phosphoinositide-specific phospholipase C. *Biochim. Biophys. Acta* 1441 255–267. 10.1016/S1388-1981(99)00150-X 10570253

[B143] Wilschut J. C. Regts J. Westenberg H. Scherphof G. (1978). Action of phospholipases A2 on phosphatidylcholine bilayers. Effects of the phase transition, bilayer curvature and structural defects. *Biochim. Biophys. Acta* 508 185–196. 10.1016/0005-2736(78)903243 565217

[B144] Winget J. M. Pan Y. H. Bahnson B. J. (2006). The interfacial binding surface of phospholipase A2s. *Biochim. Biophys. Acta* 1761 1260–1269. 10.1016/j.bbalip.2006.08.002 16962825

[B145] Yu B. Z. Berg O. G. Jain M. K. (1999a). Hydrolysis of monodisperse phosphatidylcholines by phospholipase A2 occurs on vessel walls and air bubbles. *Biochemistry* 38 10449–10456. 10.1021/bi990194z 10441140

[B146] Yu B. Z. Rogers J. Tsai M. D. Pidgeon C. Jain M. K. (1999b). Contributions of residues of pancreatic phospholipase A2 to interfacial binding, catalysis, and activation. *Biochemistry* 38 4875–4884. 10.1021/bi982215f 10200177

[B147] Yunes Quartino P. J. Barra J. L. Fidelio G. D. (2012). Cloning and functional expression of secreted phospholipases A(2) from *Bothrops diporus* (*Yarara Chica*). *Biochem. Biophys. Res. Commun.* 427 321–325. 10.1016/j.bbrc.2012.09.051 22995294

[B148] Yunes Quartino P. J. Portela M. Lima A. Duran R. Lomonte B. Fidelio G. D. (2015). A constant area monolayer method to assess optimal lipid packing for lipolysis tested with several secreted phospholipase A2. *Biochim. Biophys. Acta* 1848(10 Pt A), 2216–2224. 10.1016/j.bbamem.2015.06.003 26051123

