# Supplementary material for: Secretory Phospholipases A2 in Plants
Source: Front Plant Sci. 2019 Jul 10;10:861. doi: 10.3389/fpls.2019.00861 (PMC6635587; doi:10.3389/fpls.2019.00861)
Supplement: Supplementary file 1 [file Data_Sheet_1.pdf]

```
                   ....|....| ....|....| ....|....| ....|....| ....|....| ....|....|
                       305        315        325        335        345        355
AtsPLA2-α          KKP------- ---------- ---------- ---------- ---------- ---.......
PsPLA2             HKP------- ---------- ---------- ---------- ---------- ---.......
CssPLA2-α          HKP------- ---------- ---------- ---------- ---------- ---.......
RcsPLA2-α          HKP------- ---------- ---------- ---------- ---------- ---.......
GmsPLA2-XIB-1      HKP------- ---------- ---------- ---------- ---------- ---.......
GmsPLA2-XIB-2      HKP------- ---------- ---------- ---------- ---------- ---.......
GmsPLA2-XIB-3      HK-------- ---------- ---------- ---------- ---------- ---.......
DcsPLA2            HKP------- ---------- ---------- ---------- ---------- ---.......
NtsPLA2-II         KKP------- ---------- ---------- ---------- ---------- ---.......
LesPLA2            KKP------- ---------- ---------- ---------- ---------- ---.......
Zeamaize           HKP------- ---------- ---------- ---------- ---------- ---.......
OssPLA2-III        HKP------- ---------- ---------- ---------- ---------- ---.......
TdsPLA2-III        HKP------- ---------- ---------- ---------- ---------- ---.......
OssPLA2-II         HKRDDGQ--- ---------- ---------- ---------- ---------- ---.......
TdsPLA2-II         HKRDIGQ--- ---------- ---------- ---------- ---------- ---.......
TdsPLA2-IV         RRAPAPAPAK ---------- ---------- ---------- ---------- ---.......
OssPLA2-IV         HRP------- ---------- ---------- ---------- ---------- ---.......
AtsPLA2-δ          NIIIPKKPAS AGPVVEVDLA RSKADTKDGL GTNQGPQTKD GSKVSVPMNP SPS.......
AtsPLA2-γ          NIFNPPVLGS V-PVVEVDLA RSKVDTKDGL GTKLGLQTKE GSKVSASLNI ---.......
AtsPLA2-β          N--------- ---------- ----DMKTEL ---------- ---------- ---.......
OssPLA2-I          TQ-------- ---------- ------KLEL ---------- ---------- ---.......
TdsPLA2-I          SQ-------- ---------- ------KLEL ---------- ---------- ---.......
CssPLA2-β          GS-------- ---------- ------KFEL ---------- ---------- ---.......
NtsPLA2-I          NS-------- ---------- ------KLEL ---------- ---------- ---.......
GmsPLA2-XIA-1      D--------- ---------- ------SVPH ---------- ---------- ---.......
GmsPLA2-XIA-2      DF-------- ---------- ------SVPH ---------- ---------- ---.......
Clustal Consensus                                                            .......
```

Figure S1. Alignment of the C- terminal sequences of the plant $_{S}PLA_2$s. The GeneBank IDs of the $sPLA_2$ indicated in this figure are in the Appendix S1 at the end of Supplementary Material.

**Ats-PLA$_{2\alpha}$**	100																							
**Ats-PLA$_{2\beta}$**	56.8	100																						
**Ats-PLA$_{2\delta}$**	39.8	56.0	100																					
**Ats-PLA$_{2\gamma}$**	42.2	61.0	82.7	100																				
**NtsPLA$_2$II**	74.5	51.0	42.4	43.9	100																			
**OssPLA$_2$II**	65.4	54.2	42.9	42.8	65.0	100																		
**OssPLA$_2$I**	51.4	65.8	46.1	48.1	48.4	49.0	100																	
**DcsPLA$_2$**	74.5	52.2	42.9	44.4	77.1	63.7	45.9	100																
**ZmsPLA$_2$**	69.7	51.6	42.4	43.9	68.8	63.2	48.4	65.6	100															
**GmsPLA$_2$-XIA-I**	47.3	65.8	51.3	53.5	45.2	51.6	69.6	45.9	50.3	100														
**GmsPLA$_2$-XIA-II**	48.0	64.4	51.8	52.4	48.4	54.9	68.8	45.2	50.3	95.5	100													
**GmsPLA$_2$-XIB-I**	78.3	47.1	40.8	41.7	72.0	65.0	46.5	77.7	70.7	42.0	40.8	100												
**GmsPLA$_2$-XIB-II**	77.6	49.4	39.8	41.7	72.6	64.1	45.5	76.4	69.2	44.9	44.9	94.9	100											
**GmsPLA$_2$-XIB-III**	78.3	50.3	40.8	41.7	72.6	65.0	48.4	77.7	71.3	43.3	44.6	86.6	87.3	100										
**CssPLA$_2$-β**	31.2	40.5	39.1	39.1	34.1	29.7	40.1	31.2	29.0	37.3	35.8	32.6	31.2	31.5	100									
**CssPLA$_2$-α**	80.1	50.0	41.1	42.2	76.4	66.0	50.0	81.5	69.9	46.8	46.8	79.0	77.6	79.0	34.4	100								
**LesPLA$_2$**	73.1	51.9	42.4	44.4	90.4	65.4	48.7	74.5	70.5	48.7	49.4	73.2	75.0	74.5	33.0	76.3	100							
**NtsPLA$_2$ I**	52.7	72.1	55.0	53.5	49.7	53.4	70.1	49.7	51.5	71.4	69.4	49.7	49.4	52.2	45.5	52.6	48.1	100						
**TdsPLA$_2$-I**	50.0	67.8	47.1	49.7	47.8	49.7	89.9	48.4	49.0	66.9	69.1	47.1	45.5	49.0	40.5	51.3	48.1	71.4	100					
**TdsPLA$_2$-II**	66.2	48.4	42.4	43.9	65.0	82.8	47.1	62.4	64.3	48.4	48.4	68.2	65.6	66.2	33.7	65.6	65.0	52.9	47.1	100				
**TdsPLA$_2$-III**	66.7	48.8	36.7	39.6	67.3	60.5	46.5	66.0	82.7	47.5	47.5	67.3	64.2	69.1	33.3	69.8	67.3	49.4	48.1	61.1	100			
**TdsPLA$_2$-IV**	57.1	45.9	41.9	41.7	58.2	58.8	44.7	57.1	88.2	40.0	41.8	60.0	58.2	56.5	30.1	61.2	57.1	47.1	46.5	57.6	59.4	100		
**OssPLA$_2$-III**	69.9	52.8	42.2	42.8	69.3	62.0	49.7	66.9	83.4	49.1	49.1	66.9	66.9	73.0	33.3	72.4	66.9	54.0	50.3	64.4	81.6	62.9	100	
**OssPLA$_2$-IV**	62.2	49.4	38.7	41.7	62.8	61.6	47.0	58.5	65.2	44.5	45.1	62.2	61.0	64.6	31.2	64.0	60.4	50.6	48.2	63.4	64.0	74.1	67.7	100

**RcsPLA$_2$α**	**80.6**	**48.8**	**40.8**	**42.2**	**76.3**	**61.3**	**48.1**	**78.8**	**70.6**	**46.3**	**45.0**	**78.5**	**80.6**	**79.1**	**32.3**	**85.6**	**75.0**	**46.3**	**46.3**	**63.8**	**72.8**	**58.2**	**74.2**	**64.6**	**100**	
**PsPLA$_2$**	**78.6**	**49.7**	**41.4**	**43.9**	**77.4**	**64.8**	**47.8**	**78.0**	**71.7**	**45.9**	**45.9**	**80.5**	**79.9**	**78.6**	**31.9**	**81.1**	**76.1**	**48.4**	**47.2**	**64.2**	**72.2**	**60.0**	**73.0**	**62.2**	**83.1**	**100**

Figure S2. Data showing the percentage of similarity among the sPLA$_2$ isoforms found when comparing the sPLA$_2$s from plants, using the MatGAT v2.02 program available at http://www.angelfire.com/nj2/arabidopsis/MatGAT.html (see Campanella, J. J. et al. MatGAT: an application that generates similarity/identity matrices using protein or DNA sequences 2003, *BMC Bioinformatics* 4: 29, 1-4. DOI: 10.1186/1471-2105-4-29.

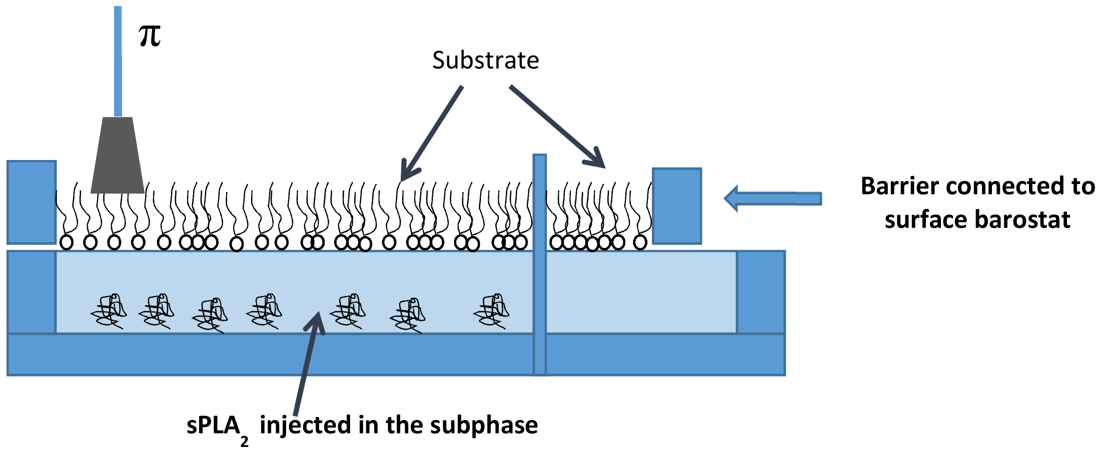

Figure S3. **Schematic representation of $sPLA_2$ hydrolysis on lipid monomolecular film in a zero order regime.** Briefly, the substrate is spread over the air/water interface at a desired surface pressure (π). The $sPLA_2$ is injected in the subphase beneath of the lipid monolayer (left compartment). After a lag time (enzyme adsorption) the hydrolysis takes place in a zero order regime. As the hydrolyzed short chain lipid is dissolved into the subphase new lipid substrate is supplied from the reservoir (right compartment) by means of a barrier connected to a surface barostat. This system keeps the surface pressure constant as the products of lipolysis are dissolved in the aqueous subphase (see Ref. [71] for more details).

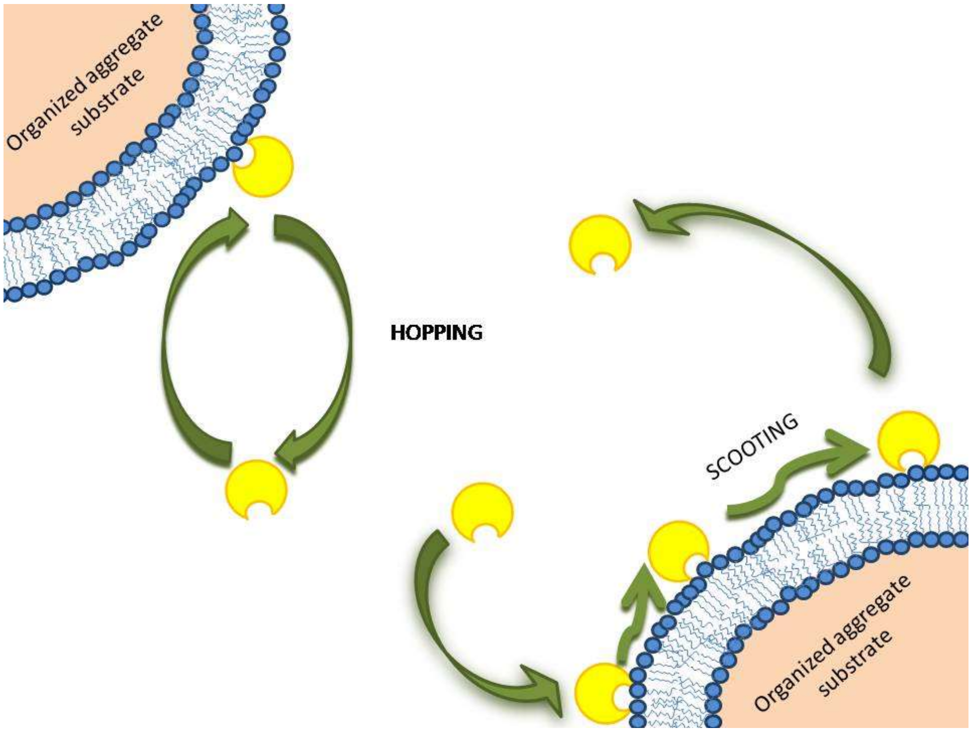

Figure S4: The two extreme mode of $sPLA_2$ catalysis at organized interfaces

Two conceptual extreme modes of $sPLA_2$ catalysis at organized interfaces. In one extreme ***“hopping”*** means that the $sPLA_2$ enzyme in each catalytic cycle (hydrolysis of a single phospholipid) desorbs and renews the cycle with a new adsorption, and continues many times in this regime. In the other extreme, in the ***“scooting”*** mode, the $sPLA_2$ enzyme remains adsorbed "walking at the surface" and hydrolyzing lipids without desorbing from the interface. (Adapted and redrawn from *Griffith and Ryan*, *Biochim. Biophys. Acta 1999, 1441, 237-254)*

```
CssPLA2β          MRPIHAHFQRIKVAIILYFHLLERLLISNDNSHSMMACLQLACVLSCRRCHVAHWHSLDR
NtsPLA2I          -------------------------MQGGDNS----------------------------
Luspla2-II        -------------------------MRFNGLA----------------------------
OssPLA2I          ---------------------------MP-------------------------------
TdsPLA2I          -------------------------MAMA-------------------------------
AtsPLA2-δ         ------------------------------------------------------------
AtsPLA2-γ         ------------------------------------------------------------
AtsPLA2-β         ------------------------------------------------------------
GmsPLA2-XIA-I     ------------------------------------------------------------
GmsPLA2-XIA-II    ------------------------------------------------------------
LusPLA2-I         ------------------------------------------------------------
DcsPLA2           ------------------------------------------------------------
GmsPLA2-XIB-I     ------------------------------------------------------------
GmsPLA2-XIB-II    ------------------------------------------------------------
GmsPLA2-XIB-III   ------------------------------------------------------------
CssPLA2α          ------------------------------------------------------------
RcsPLA2α          ------------------------------------------------------------
AtsPLA2-α         ------------------------------------------------------------
PsPLA2            ------------------------------------------------------------
NtsPLA2II         ------------------------------------------------------------
LesPLA2           ------------------------------------------------------------
OssPLA2III        ------------------------------------------------------------
ZmsPLA2           ------------------------------------------------------------
TdsPLA2III        ------------------------------------------------------------
OssPLA2II         ------------------------------------------------------------
TdsPLA2II         ------------------------------------------------------------
TdsPLA2IV         ------------------------------------------------------------
NnsPLA2           ------------------------------------------------------------
PpsPLA2           ------------------------------------------------------------


CssPLA2β          FSLFRQRRLLCHSLSVVQSSCIVGQIQLSLPKGILVILINFFVNKQFFLSTQGKQTRRVE
NtsPLA2I          ----------------LTSSFILTTLVFS-----------------FFL-----------
Luspla2-II        -----------------AAFTFVFLFLFS-------------------------------
OssPLA2I          -----------------PRSPLLALVFLA-------------------------------
TdsPLA2I          -----------------MAMTVLLVVLLL-------------------------------
AtsPLA2-δ         ----------------MIRGGALTHVALG-------------------------------
AtsPLA2-γ         ----------------MITGLALSRVAFG-------------------------------
AtsPLA2-β         ----------------MMFRTSLMRFAAAF------------------------------
GmsPLA2-XIA-I     -----------------MSRAATSFGILLC------------------------------
GmsPLA2-XIA-II    -----------------MSRAAASFGILLC------------------------------
LusPLA2-I         --MAASSGIPRHHQYHLSIHHLVLLLLLLVSLC---------------------------
DcsPLA2           --------MVFLKFVYLALSYVFIICLLNFNG----------------------------
GmsPLA2-XIB-I     ------MVPTPQLKYVLLLFYCTF-AFNLLS-----------------------------
GmsPLA2-XIB-II    ------MVPAPSFKYVLF-FCCTF-AFNLLS-----------------------------
GmsPLA2-XIB-III   ------MVPSQLSKYGLLFISCTFFLINFLT-----------------------------
CssPLA2α          ------MVMTHQHSLKLAIAHVFIFLILSVS-----------------------------
RcsPLA2α          -------MANLSQPLNLVAYLFFFCFLLSLSFP---------------------------
AtsPLA2-α         ------MAAP----------IILFSFLLFFSV----------------------------
PsPLA2            ------MAAYKSCHLSLKFVLLISSFVLYFSCP---------------------------
NtsPLA2II         --------MAFLQSLKFSLQLLSFCIIALR------------------------------
LesPLA2           -------------MVKLSLHFLAFCIIAIFTNLF--------------------------
OssPLA2III        ------MA--RGGS---FSRLRLRAGVVVAAAAAALLLFA--------------------
ZmsPLA2           ------ME--RGSS---WRLPAVVVGILVCAA----------------------------
TdsPLA2III        ------MAHGRGGSRRLHARLLAVVGLLACAA----------------------------
OssPLA2II         ----------MRFFLKLAPRCSVLLLLLLVT-----------------------------
TdsPLA2II         ----------MRSVLGLSHWCSLLLLLSLVT-----------------------------
TdsPLA2IV         ----------------MHTGRLLPLLLLLAA-----------------------------
NnsPLA2           ------------------MNPAHLLILAA-------------------------------
PpsPLA2           ------------------MKFLVLAVLLT-------------------------------
```

```
CssPLA2β            DRREDGKREKKMLLGAFVPFRTCVIAAFAFVFIIVFSESASALNDS------------QV
NtsPLA2I            -----------------------------------FAIAESTNNSQ------------GV
Luspla2-II          ----------------------------------LVSSSATNRHGA------------QA
OssPLA2I            -----------------------------------AGVLSSATSPP------------PP
TdsPLA2I            -----------------------------------AGG-SAAASPP------------PP
AtsPLA2-δ           ----------------------------------LTVFLLLAVVHS------------QE
AtsPLA2-Y           ----------------------------------LTAFLLLAVVSS------------QE
AtsPLA2-β           ---------------------------------FAIVFVVLVGVAR------------SE
GmsPLA2-XIA-I       ---------------------------------LFLAAAAVVNCSD------------QA
GmsPLA2-XIA-II      ---------------------------------LLLLVAA-VNCSD------------QG
LusPLA2-I           --------------------------------LTAVDGLNLGVQAT--DQPGVTL---SK
DcsPLA2             ---------------------------------ISVYALNIGVQSF--DAS-VQL---SK
GmsPLA2-XIB-I       ---------------------------------TPACALNIGAETT--GVA-VSV---SK
GmsPLA2-XIB-II      ---------------------------------TPVRALNIGAETT--GVA-VSV---GK
GmsPLA2-XIB-III     ---------------------------------IPISSLNIGVETT--GIT-VSV---SK
CssPLA2α            -----------------------------------VHALNIGVQSA--YSA-ISV---SK
RcsPLA2α            --------------------------------STPVHALNIGVQTA--NSA-ITL---SK
AtsPLA2-α           ----------------------------------SVSALNVGVQLI--HPS-ISL---TK
PsPLA2              ----------------------------------SVYALNVGVQTT--GTG-VSL---MK
NtsPLA2II           ------------------------------FSPISIHALNIGIET---NAG-ISL---EK
LesPLA2             ------------------------------NSPISIHALNVGVET---NAG-LSL---EK
OssPLA2III          ------------------------------VVAPPAAALNIGLQS---AGDGASK---AG
ZmsPLA2             ------------------------------LFSPPAAALNIGIQS---AGDGVSK---QQ
TdsPLA2III          -------------------------------VAPRSSALNVGLQTLDADGDGVSK---QQ
OssPLA2II           ----------------------------------ASRGLNIGDLL----GSTPAK---DQ
TdsPLA2II           ----------------------------------ASRGLEVGDLFS--HGKPPAG---KQ
TdsPLA2IV           ----------------------------------AGRSLARGGIFG--AASPPSSPDGDQ
NnsPLA2             -----------------------------------VCVSPLGASSNRPMPLNLYQFKNMV
PpsPLA2             -----------------------------------VGAAQEGISSR-----ALWQFRSMI


CssPLA2β            KCSRTCVAENCN----SVGIRYGKYCGVGWSGCPGEKPCDDLDACCKIHDECVDKK----
NtsPLA2I            RCSKTCVAENCN----SIGIRYGKYCGVGWSGCPGEKPCDDLDTCCKIHDECVEKN----
Luspla2-II          ICSRTCVVQNCD----SFGIRYGKYCGLGWTGCPGEKPCDDLDACCKIHDDCVGKN----
OssPLA2I            PCSRSCAALNCD----SVGIRYGKYCGVGWSGCDGEEPCDDLDACCRDHDHCVDKK----
TdsPLA2I            PCSRSCATLNCD----SVGIRYGKFCGVGWSGCEGEEPCDDLDACCRDHDHCVGKK----
AtsPLA2-δ           KCSKTCIAQKCN----VLGIRYGKYCGIGYFGCPGEPPCDDLDDCCMTHDNCVDLK----
AtsPLA2-Y           KCSNTCIAQNCN----SLGIRYGKYCGIGYFGCPGEPPCDDLDACCMTHDNCVDLK----
AtsPLA2-β           ECTRTCIAQNCD----TLSIRYGKYCGIGHSGCPGEEPCDDLDACCKIHDHCVELN----
GmsPLA2-XIA-I       NCSTTCIAEQCD----TVGIKYGKYCGVGYWGCAGEKPCDDLDACCMAHDDCVDKF----
GmsPLA2-XIA-II      NCSTTCIVEQCD----TIGIKYGKYCGVGYWGCAGEKPCDDLDACCMAHDNCVDKF----
LusPLA2-I           ECSRKCESAFCA---VPPLLRYGKYCGLLYSGCPGETPCDGLDSCCMNHDLCVQSKN---
DcsPLA2             ECSRKCESEFCS---LPPLLRYGKYCGLLYSGCPGEMPCDGLDACCMSHDACVQSKG---
GmsPLA2-XIB-I       ECSRQCESSFCS---VPPLLRYGKYCGLLYSGCPGERPCDGLDACCMKHDQCVSAKN---
GmsPLA2-XIB-II      ECSRQCESSFCS---VPPLLRYGKYCGLLYSGCPGERPCDGLDACCMKHDQCVSAKN---
GmsPLA2-XIB-III     ECSRTCESSFCS---VPPLLRYGKYCGLLYSGCPGEKPCDGLDACCMYHDKCVQAKN---
CssPLA2α            DCSRKCESDFCS---VPPFLRYGKYCGLLYSGCPGEKPCDGLDACCMKHDACVQAKN---
RcsPLA2α            ECSRKCESEFCS---VPPFLRYGKYCGLLYSGCPGEKPCDGLDACCMKHDSCVQAKN---
AtsPLA2-α           ECSRKCESEFCS---VPPFLRYGKYCGLLYSGCPGERPCDGLDSCCMKHDACVQSKN---
PsPLA2              ECSRKCESEFCS---VPPFLRYGKYCGLLYSGCPGERPCDGLDACCMKHDVCIQLKN---
NtsPLA2II           ECSRTCESKFCA---VPPFLRYGKYCGVLYSGCPGEQPCDGLDACCMKHDLCIQRKG---
LesPLA2             ECSRTCESKFCA---VPPFLRYGKYCGIMYSGCPGEQPCDALDACCMKHDLCIQHKD---
OssPLA2III          LCSRTCESDHCT---TPPLLRYGKYCGILYSGCPGEQPCDELDACCMHHDNCVQAK----
ZmsPLA2             ACSRTCESDHCT---TPPFLRYGKYCGILYSGCPGEPPCDALDACCMHHDNCVQAK----
TdsPLA2III          ACSRTCESDHCT---TAPFLRYGKYCGILYSGCPGERPCDPLDACCMHHDNCVLVK----
OssPLA2II           GCSRTCESQFCT---IAPLLRYGKYCGILYSGCPGERPCDALDACCMVHDHCVDTHN---
TdsPLA2II           DCSRTCESKFCT---VPPVLRYGKYCGILYSGCPGEKPCDALDACCMVHDHCVAANN---
TdsPLA2IV           KCSRTCESAYCTGTLEAPLMRYGKYCGVSYTGCPGEPPCDALDACCMLHDACVQAT----
NnsPLA2             QCTVP-NRSWWD------FADYGCYCGRGGSGTP----VDDLDRCCQVHDNCYGEAEKIS
PpsPLA2             KCAIPGSHPLMD------FNNYGCYCGLGGSGTP----VDELDRCCETHDNCYRDAKNLD
                     *:                  ** :**    *       * ** **  ** *
```

```
CssPLA2β               -----------GLTNIKCHEKFKRCIKK----------VQKSAK------VGFSRECPYD
NtsPLA2I               -----------GMTNVKCHEKFKRCIKK----------VQKSRK------AGFSRECPYD
Luspla2-II             -----------GLTNIKCHEKFKKCAKK----------VQNSGK------IGFSKRCSYE
OssPLA2I               -----------GLMSVKCHEKFKNCMRK----------VKKAGK------IGFSRKCPYE
TdsPLA2I               -----------GLMSIKCHEKFKNCMRK----------VKKAGK------VGFSTKCPYE
AtsPLA2-δ              -----------GMTYVDCHKQFQRCVNE----------LKQSIQESNNQKVGFSKECPYS
AtsPLA2-Y              -----------GMTYVNCHKQFKRCVNK----------LSKSIKHSNGEKIGFSTQCPYS
AtsPLA2-β              -----------GMTNISCHKKFQRCVNR----------LSKAKQ-SKNKKVGFSTKCPYS
GmsPLA2-XIA-1          -----------GMTHVKCHKKLKNCLTR----------ELKSGK------VGFSKECPYS
GmsPLA2-XIA-2          -----------GMTHVKCHKRLKNCLTR----------ELKSGK------VGFSKECPYS
LusPLA2-I              ----------NDYLSKECSEKLVNCMKN----------FIKSGGHSFK-----GSTCDAT
DcsPLA2                ----------DDYLSQECSNKLISCMEN----------FKAHGAHTFK-----GSTCQAD
GmsPLA2-XIB-1          ----------NDYLSQECSQTFINCMNN----------FKNSRAPTFK-----GNTCDAD
GmsPLA2-XIB-2          ----------NDYLSQECSQTFINCMNN----------FKNSKAPTFK-----GNTCDVD
GmsPLA2-XIB-III        ----------NDYLSQECSQTFINCMQK----------FKNSRAPTFK-----GNACQVD
CssPLA2α              ----------NDYLSQECSKNFIDCMEK----------FKRAGGRSFK-----GNTCGVE
RcsPLA2α               ----------NDYLSQECSQNFINCMND----------FKNKGGHTFK-----GSKCQVD
AtsPLA2-α              ----------NDYLSQECSQKFINCMNN----------FSQKKQPTFK-----GNKCDAD
PsPLA2                 ----------NDYLSEECSQTFLNCMKN----------FKDSGGRTFK-----GNKCDVG
NtsPLA2II              ----------NNYLNLECNQNFLNCVAT----------FTKSGAPSFK-----GNTCSVG
LesPLA2                ----------NNYLNLECNENFLSCVAK----------FTKSGSPTFK-----ENTCSIT
OssPLA2III             ----------NDYLSTACNEELLECLAR----------LRE-GSSTFQ-----GNKCMID
ZmsPLA2              ----------MDYLSTACNEALLDCLAR----------LRE-GTSTFN-----GNKCMIV
TdsPLA2III             ----------NDYLSTECNEGLLECLAE----------LRD-GTGTFE-----GNKCMID
OssPLA2II              ----------DDYLNTMCNENLLSCID-----------RVS--GATFP-----GNKCNVG
TdsPLA2II              ----------NDYLNTGCNENLLGCLD-----------GVNPAGPTFP-----GNKCGVG
TdsPLA2Iv              ----------DDYLNMWCNQSLLDCVAAVRTAAAAAGGGVEAVLTTFE-----GNSCNAT
NnsPLA2                RC-----WPYFKTYSYECSQGTLTCKGG-----------NNACAAAVCDCDRLAAICFAG
PpsPLA2                SCKFLVDNPYTESYSYSCSNTEITCNSK-----------NNACEAFICNCDRNAAICFSK
                                        * :    *                               *

CssPLA2β               TVVPTMVQGMDMAILLSQLGG------------------------SKFEL----------
NtsPLA2I               VAVPTMVQGMDMAIMFSQLGN------------------------SKLEL----------
Luspla2-II             KAIPTMLNGIDMGILLSQFGD------------------------SKPEL----------
OssPLA2I               MAMATMTSGMDMAIMLSQLGT------------------------QKLEL----------
TdsPLA2I               MAMATMTQGMDMAIMLSQLGS------------------------QKLEL----------
AtsPLA2-δ              TVIPTVYRGMNYGIFFSGIGNIIIPKKPASAGPVVEVDLARSKADTKDGLGTNQGPQTKD
AtsPLA2-Y              IVIPTVFNGMDYGIFFSGIGNIFNPPVLGSV-PVVEVDLARSKVDTKDGLGTKLGLQTKE
AtsPLA2-β              VVIPTVNQGMDIGILFSQLGN-----------------------DMKTEL----------
GmsPLA2-XIA-1          RAAPTMIRGMDLAILLSQLGD-----------------------SVPH------------
GmsPLA2-XIA-2          RAAPTMIRGMDLAILLSQLGDF----------------------SVPH------------
LusPLA2-I              DVIDVISLVMDAALLAGRYLHRP-------------------------------------
DcsPLA2                DVIQVIKFVMEAAILAGRVLHKP-------------------------------------
GmsPLA2-XIB-1          DVIEVIHVVMEAALLAGRVLHKP-------------------------------------
GmsPLA2-XIB-2          DVIEVIHVVMEAALLAGRVLHKP-------------------------------------
GmsPLA2-XIB-3          DVIEVINVVMEAALLAGRVLHK--------------------------------------
CssPLA2α               EVIDVIIVVMEAALLAGRVLHKP-------------------------------------
RcsPLA2α               EVIDVISVVMEAALIAGRYLHKP-------------------------------------
AtsPLA2-α              EVIDVISIVMEAALIAGKVLKKP-------------------------------------
PsPLA2                 EVVDIISLVIDAALLAGKVLHKP-------------------------------------
NtsPLA2II              TVVRVITDVIDAAVVAGNIFKKP-------------------------------------
LesPLA2                TVVRVITDVIDAAVAAGKIFKKP-------------------------------------
OssPLA2III             EVIDVISLVIEAAVVAGRLLHKP-------------------------------------
ZmsPLA2                EVIDVISLVIEAAVVAGRVLHKP-------------------------------------
TdsPLA2III             EVIDVITVVIEAAVVAGRVLHKP-------------------------------------
OssPLA2II              QTASVIRGVIETAVFAGKILHKRDDGQ---------------------------------
TdsPLA2II              ETAFVIKGVIEAAVLAGKILHKRDIGQ---------------------------------
TdsPLA2Iv              DVADEITSILEAAVYAGSILRRAPAPAPAK------------------------------
NnsPLA2                APYNDNNYNIDLKARCQ-------------------------------------------
PpsPLA2                APYNKEHKNLDTKKYC--------------------------------------------
                                ::
```

```
CssPLA2β          -------------
NtsPLA2I          -------------
Luspla2-II        -------------
OssPLA2I          -------------
TdsPLA2I          -------------
AtsPLA2-δ         GSKVSVPMNPSPS
AtsPLA2-γ         GSKVSASLNI---
AtsPLA2-β         -------------
GmsPLA2-XIA-1     -------------
GmsPLA2-XIA-2     -------------
LusPLA2-I         -------------
DcsPLA2           -------------
GmsPLA2-XIB-1     -------------
GmsPLA2-XIB-2     -------------
GmsPLA2-XIB-3     -------------
CssPLA2α          -------------
RcsPLA2α          -------------
AtsPLA2-α         -------------
PsPLA2            -------------
NtsPLA2II         -------------
LesPLA2           -------------
OssPLA2III        -------------
ZmsPLA2           -------------
TdsPLA2III        -------------
OssPLA2II         -------------
TdsPLA2II         -------------
TdsPLA2Iv         -------------
NnsPLA2           -------------
PpsPLA2           -------------
```

Figure S5 Complete amino acid sequence of the $sPLA_2$ reported for plants. The alignment of the sequences shows the putative cleavage sites (in yellow) of signal peptidase splitting the signal sequence and the mature form of $sPLA_2$. For comparison, two $sPLA_2$s from animals are also shown: *Naja naja* (from cobra venom) and from pig pancreatic (the latter the mature form is as a zymogen pre-protein). Signal peptide was determined by using signalP 3.0 server (http://www.cbs.dtu.dk/services/SignalP-3.0/).

Appendix S1. Information attached of the accession numbers of all $sPLA_2$ reported and used in Fig. 3 and 4 of the paper and in Fig. S1 of Supplementary Material.

The GenBank ID are as follows: *A. thaliana* isoform α (AtsPLA2α), β (*At*sPLA2β), γ (*At*sPLA2γ) and δ (*At*sPLA2δ), AY136317,AF541915, AY148346 and AY148447, respectively; carnation *D. caryophillus* (*Dc*sPLA2), AF064732;maize *Z. maize Zm*$sPLA_2$ ACG40877; castor bean *R. communis* (*Rc*$sPLA_2$α) XP_002523659; wheat *T. durum* isoform I (*Td*sPLA2I), II (*Td*sPLA2II), III (*Td*sPLA2III) and IV (*Td*sPLA2IV), JX021445, JX021446, JX021447and JX0214458, respectively; orange C. *sinensis* isoform α (*Cs*sPLA2α) and β (*Cs*sPLA2β), ADF55749.1 and ADF55750, respectively; rice *O. sativa* isoform I (*Os*sPLA2I), II (*Os*sPLA2II), III (*Os*sPLA2III) and IV (*Os*sPLA2IV), AJ238116, AJ238117, BAS85989 and BAT14386, respectively; tobacco *N. tabaccum* isoform I (*Nt*sPLA2I) and II (*Nt*sPLA2II), AB190177 and AB190178, respectively; tomato *L. esculentum* (*Le*sPLA2), AI487873 and opium *P. somniferum* ANY57826; flax *L. usitatissimun* isoform I ANP93618 and II ANP93619, while for the isoforms of $sPLA_2$ soybean *G.max* (*Gm*$sPLA_2$-XIA-I, -2 and *Gm*$sPLA_2$-XIB-I, -II, and -III) are ACU16523, ACU18951, ACU19490, ACU15282 and ACU24341, respectively. For animals $sPLA_2$ was *N. naja Nn*$sPLA_2$ AAA66029 and pig pancreatic *S. scrofa Pp*$sPLA_2$ AAA31101.
